# Synergistic Activation of Antitumor Immunity by a Particulate Therapeutic Vaccine

**DOI:** 10.1002/advs.202100166

**Published:** 2021-04-15

**Authors:** Junhua Mai, Zhaoqi Li, Xiaojun Xia, Jingxin Zhang, Jun Li, Haoran Liu, Jianliang Shen, Maricela Ramirez, Feng Li, Zheng Li, Kenji Yokoi, Xuewu Liu, Elizabeth A. Mittendorf, Mauro Ferrari, Haifa Shen

**Affiliations:** ^1^ Department of Nanomedicine Houston Methodist Academic Institute Houston TX 77030 USA; ^2^ Xiangya Hospital of Central South University Changsha Hunan 410000 China; ^3^ Department of Experimental Medicine Sun Yat‐sen University Cancer Center State Key Laboratory of Oncology in South China Guangzhou 510060 China; ^4^ School of Ophthalmology & Optometry School of Biomedical Engineering Wenzhou Medical University Wenzhou 325035 China; ^5^ Center for Bioenergetics Houston Methodist Academic Institute Houston TX 77030 USA; ^6^ Department of Surgery Brigham and Women's Hospital Boston MA 02115 USA; ^7^ Breast Oncology Program Dana‐Farber/Brigham and Women's Cancer Center Boston MA 02115 USA; ^8^ Department of Pharmaceutics School of Pharmacy University of Washington Seattle WA 98195 USA; ^9^ Houston Methodist Cancer Center Houston TX 77030 USA; ^10^ Department of Cell and Developmental Biology Weill Cornell Medical College New York NY 10065 USA

**Keywords:** antigen peptides, cancer, innate immunity, microparticles, nanoparticles, therapeutic vaccines

## Abstract

Success in anticancer immune therapy relies on stimulation of tumor antigen‐specific T lymphocytes and effective infiltration of the T cells into tumor tissue. Here, a therapeutic vaccine that promotes proliferation and tumor infiltration of antigen‐specific T cells in both inflamed and noninflamed tumor types is described. The vaccine consists of STING agonist 2′3′‐cGAMP, TLR9 ligand CpG, and tumor antigen peptides that are loaded into nanoporous microparticles (*μ*GCVax). *μ*GCVax is effective in inhibiting lung metastatic melanoma, primary breast cancer, and subcutaneous colorectal cancer in their respective murine models, including functional cure of HER2‐positive breast cancer. Mechanistically, *μ*GCVax potently stimulates type I interferon expression in dendritic cells, and promotes CD8^+^ and CD103^+^ dendritic cell maturation and migration to lymph nodes and other lymphatic tissues. Antitumor responses are dependent on TLR9 and interferon *α*/*β* receptor signaling, and to a less extent on STING signaling. These results demonstrate a high potential for *μ*GCVax in mediating antitumor immunity in personalized cancer therapy.

## Graphical abstract

Adjuvant activity is key for potency of a therapeutic cancer vaccine. In this article, a combination of soluble and particulate adjuvants is described that synergistically and potently stimulates dendritic cells by promoting type I interferons and other inflammatory cytokines, leading to robust activation of antigen‐specific cytotoxic T cells and consequent antitumor immunity from the vaccine.

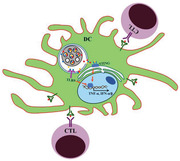

## 1 Introduction

Cancer immunotherapy has achieved unprecedented clinical efficacy in multiple cancer types.^[^ ^1^, ^2^, ^3^, ^4^ ^]^ However, only a small portion of cancer patients can benefit from the success, and many patients fail to respond and mount effective antitumor immune responses.^[^ ^5^, ^6^ ^]^ The underlying mechanisms for this failure are complex, with one major consideration being the immune‐suppressive tumor microenvironment.^[^ ^7^, ^8^ ^]^ Multiple lines of evidence have shown that the presence of tumor‐infiltrating lymphocytes (TIL) serves as a prognostic marker and predicts response to different therapies including immunotherapy and chemotherapy. Pathologic and genomic studies revealed that tumor infiltration of CD8^+^ T lymphocytes and tumor immunogenicity generally correlate with effective therapy response.^[^ ^9^, ^10^ ^]^ Tumors lacking TIL have been characterized as “noninflamed,” and generally correlate with treatment failure and poor prognosis.^[^ ^11^ ^]^ For example, the efficacy of checkpoint blockade antibody in patients with breast cancer, which has relatively less TIL is far less effective compared to that in patients with melanoma or nonsmall cell lung carcinoma, tumor types with abundant TIL that are characterized as “inflamed.”^[^ ^12^ ^]^ Thus, how to promote T cell infiltration and maintain function of T cells in the tumor microenvironment is a focus for developing effective immunotherapy, especially for the noninflamed tumor types.^[^ ^13^ ^]^

Innate immune recognition of cancer is a critical step for spontaneous tumor‐specific T cell priming and subsequent T cell infiltration.^[^ ^14^ ^]^ Antigen presenting cells, mainly dendritic cells (DCs), capture tumor‐derived antigens and danger signal molecules, and process antigens to form antigen epitope‐MHC complexes which are then presented to T cells and activate these cells together with co‐stimulation signals on DC cell surface.^[^ ^15^ ^]^ Stimuli such as pathogen‐associated molecular patterns (PAMPs) from invading microbes or danger‐associated molecular patterns released from dying tumor cells can bind to and activate pattern recognition receptors (PRRs) on DCs. This in turn promotes DC activation and primes appropriate T cell responses, thereby bridging innate and adaptive immunity.^[^ ^14^, ^16^ ^]^ Effective T cell priming requires not only specific TCR‐antigen recognition and co‐stimulation signals, but also T cell‐activating cytokines from DCs.^[^ ^17^ ^]^ Type I interferons (IFN‐I) and inflammatory cytokines have been shown to be critical both for DC maturation and for effective T cell priming.^[^ ^12^ ^]^ These immune‐activating cytokines can be induced from innate immune receptor activation by tumor‐derived ligands or artificially administrated adjuvants. Indeed, intratumoral administration of the Toll‐like receptor 9 (TLR9) ligand CpG oligonucleotide (CpG) or stimulator of interferon genes (STING) agonist 5,6‐dimethylxanthenone‐4‐acetic acid (DMXAA) can elicit strong antitumor immunity by promoting T cell priming and tumor killing while relieving immune suppression.^[^ ^18^, ^19^ ^]^

Therapeutic cancer vaccines can effectively boost cancer immune recognition and promote antitumor immunity. To facilitate DC maturation and effective T cell priming, vaccines often contain soluble or particulate adjuvants that stimulate innate immunity, promote antigen presentation, and induce co‐stimulation signals and helper cytokines.^[^ ^20^ ^]^ Many types of PAMPs including TLR ligands, NOD‐like receptor ligands, and RIG‐I‐like receptor ligands have been evaluated for their antitumor potency.^[^ ^21^ ^]^ Although aluminum salt (alum), a particulate adjuvant that activates the inflammasomes,^[^ ^22^, ^23^ ^]^ is the most common particulate adjuvant for human vaccines, its application in therapeutic cancer vaccine development has been unsuccessful so far, mainly due to its preference to stimulate a Th2‐biased immune responses.^[^ ^24^ ^]^ We have previously identified nanoporous silicon microparticle (*μ*‐particles) as a new class of particulate adjuvants for therapeutic DC vaccine. The *μ*‐particles stimulate a mild but significant level of IFN‐I response in DCs by activating TRIF‐ and MAVS‐dependent pathways, and exhibit prolonged early endosome localization which promotes antigen processing and cross‐presentation.^[^ ^25^ ^]^ Interestingly, several innate immune receptors are either endosomal proteins, such as TLR9, or associated with endosomes dependent on activation status, such as STING.^[^ ^26^ ^]^ We hypothesized that inclusion of stimulating ligands into the microparticles could facilitate their binding to innate immune receptors on early endosomes in antigen presenting cells, thereby inducing stronger helper cytokine production and subsequent T cell priming. To this end, we screened a group of ligands in different innate immune response pathways, and identified a unique combination of a TLR9 ligand and a STING ligand that promoted DC activation. The *μ*‐particles were required not only for efficient antigen presentation, but also for the synergistic effect of innate immune activation. A particulate vaccine was prepared by loading the TRL9 ligand CpG, STING agonist cyclic 2′3′‐GAMP (cGAMP), and tumor antigen into *μ*‐particles. This vaccine (*μ*GCVax) elicited high levels of IFN‐I and inflammatory cytokine production in DCs and induced strong T cell‐mediated antitumor immunity not only in the inflamed metastatic melanoma but also in noninflamed breast and colorectal cancers.

## 2 Results

### 2.1 Composition of a Particulate Adjuvant

**Figure 1:**
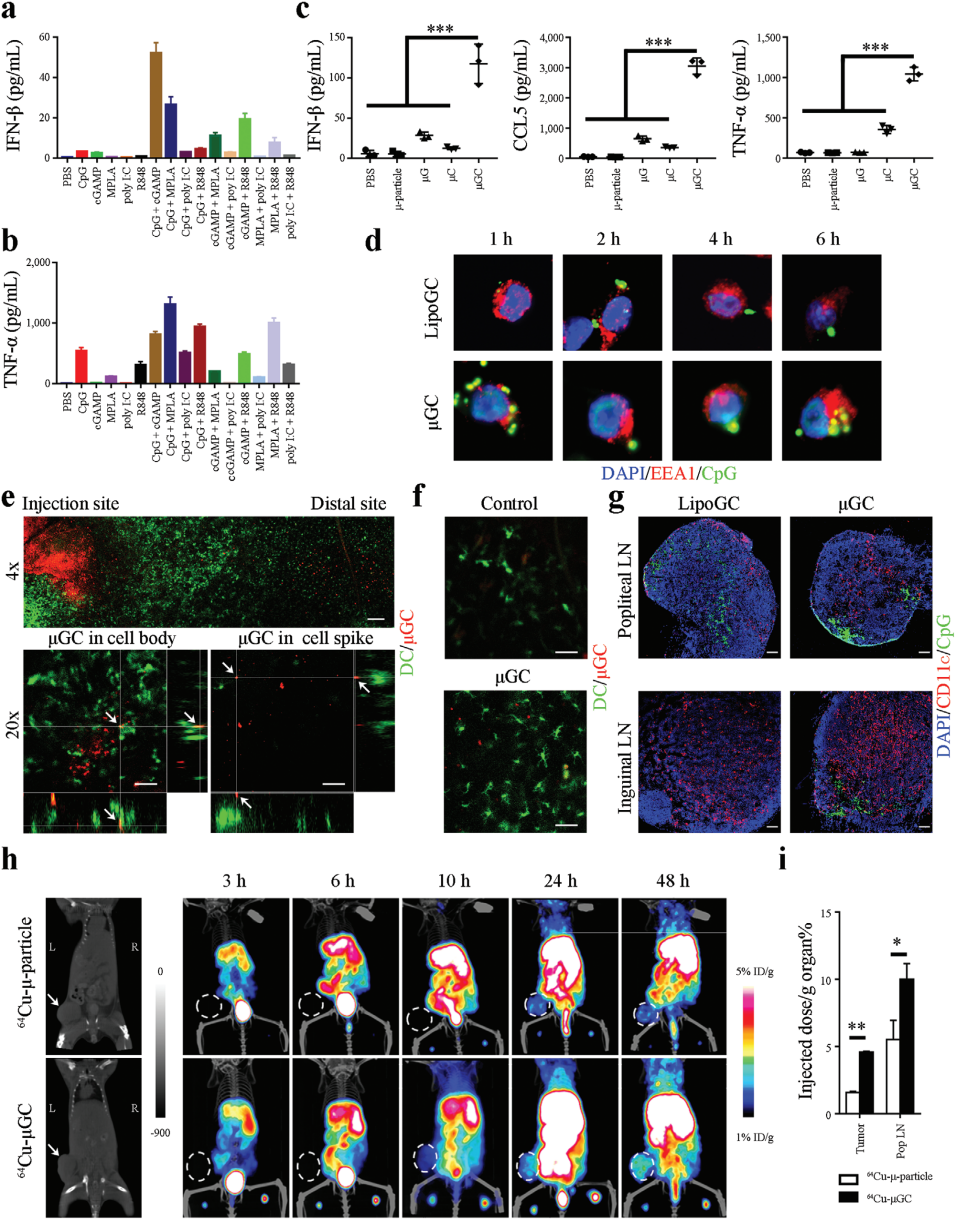
DC activation by adjuvants in vitro and in vivo. a,b) Activation of IFN‐*β* and TNF‐*α* expression in BMDC by soluble TLR ligands and a STING agonist. BMDCs were treated with a TLR ligand, a STING agonist, or their combination for 24 h, and enzyme‐linked immunosorbent assay (ELISA) was applied to measure a) IFN‐*β* and b) TNF‐*α* levels in cell growth media. Samples were triplicated. Error bars: mean +/− SD ***: *p* < 0.001. c) Synergistic activation of cytokine expression in BMDCs treated with *μ*GC particles. The *μ*‐particles loaded with liposomal cGAMP (*μ*G), CpG (*μ*C), or cGAMP and CpG combination (*μ*GC) were applied to treat BMDCs, and levels of IFN‐*β*, CCL‐5, and TNF‐*α* in cell growth media were determined with ELISA 24 h after treatment. Samples were triplicated. Error bars: mean +/− SD. d) Confocal microscopic analysis on time‐dependent subcellular localization of LipoGC and *μ*GC in dendritic cells. DC2.4 cells were treated with FITC‐labeled LipoGC or *μ*GC (in green) for up to 6 h and stained with DAPI for nuclei (in blue) and an anti‐EEA1 antibody for early endosomes (in red). e) Intravital microscopic image of EYFP‐expressing DCs (in green) adjacent to the site of *μ*GC (in red) injection. Upper panel: Overview of image. Bottom panel: Z‐stack imaging of focused areas displaying *μ*GC particles (in red, pointed with white arrows) inside the cell body (left) or in the spike (right) of DCs (in green). f) Morphology of DCs adjacent to the injection site in mice treated with control PBS or *μ*GC. g) Microscopic analysis of LipoGC and *μ*GC in lymph nodes. Mice with primary TUBO tumors were inoculated with FITC‐labeled LipoGC or *μ*GC in the foot pads, and popliteal and inguinal lymph nodes were collected 24 h later. Frozen sections of lymph nodes were stained with DAPI (in blue) and anti‐CD11c antibody (in red). Bar: 100 µm. h) PET‐CT tracking of particle transport in mice with primary TUBO tumors. Mice were inoculated with ^64^Cu‐labeled *μ*‐particles or *μ*GC in the foot pads, and time‐dependent particle transport was monitored with PET‐CT imaging in the next 48 h. Primary tumors were pointed with arrows in the left panel and circled in rest panels. Representative graphs are shown. *n* = 3 mice per group. i) Quantitative analysis of radiation activities in tumor and popliteal lymph nodes (Pop LN) was measured and compared. Statistics: ANOVA for multi‐group comparison and Student's *t‐*test for comparison between two groups. *n* = 3 mice per group. Error bars, mean +/− SD. *: *p* < 0.05; **: *p* < 0.01.

DCs express a set of TLRs and STING, although expression levels varied dependent on the cell origin (Figure S1, Supporting Information). For an example, the GM‐CSF and IL‐4‐induced bone marrow‐derived dendritic cells (GM‐CSF/IL‐4‐BMDCs) had a comparable level of STING expression with Flt3 ligand‐induced DCs and splenic DCs, but showed lower TLR9 expression. In addition, TLR3 expression was barely detectable in GM‐CSF/IL‐4‐BMDCs or splenic DCs, but was moderate in Flt3 ligand (Flt3L)‐induced CD8^+^ DCs and plasmacytoid DCs (pDCs). We treated GM‐CSF/IL‐4‐BMDCs with individual TLR ligands and cGAMP to identify combinations that might synergize IFN‐I expression. Dosages were selected such that only a minimal level of IFN‐*β* production was triggered by a single agent so that any synergistic activation could be easily detected. Combining CpG with cGAMP or the TLR4 ligand monophosphoryl lipid A (MPLA) drastically raised IFN‐*β* levels in cell growth media. A similar pattern was observed in cells treated with cGAMP and the TLR7 agonist R848 (**Figure** **1**a). The results correlated well with expression pattern of the respective receptors (Figure S1, Supporting Information). In comparison to IFN‐*β* stimulation, single agent CpG could trigger GM‐CSF/IL‐4‐BMDCs to secrete TNF‐*α*, and combinations of CpG/cGAMP, CpG/MPLA, CpG/R848 further elevated TNF‐*α* levels. MPLA/R848 combination could also trigger potent TNF‐*α* expression, although none of them were very effective as single agents (Figure 1b). The different expression pattern between IFN‐*β* and TNF‐*α* is in line with the expectation that IFN‐I and inflammatory cytokines are differentially regulated in response to PRR activation.^[^ ^27^ ^]^ Interestingly, IL‐12p70 expression did not correlate well with IFN‐*β* or TNF‐*α*, and CpG alone could promote strong cytokine expression (Figure S2, Supporting Information).

The *μ*‐particles were manufactured to comprise 40–100 nm size pores (Figure S3a, Supporting Information) that could be loaded with nanoparticles such as liposomes and polymeric drugs.^[^ ^28^, ^29^, ^30^ ^]^ We loaded liposomes encapsulated with a single agent or combined TLR ligands and cGAMP into the *μ*‐particles (Figure S3b, Supporting Information). Particles that passed quality controls were applied for biological assays (Figure S3c, Supporting Information). Incubation of BMDCs with liposomal cGAMP or CpG single agent‐loaded *μ*‐particles (*μ*G and *μ*C) mildly stimulated IFN‐*β* expression, while treatment with cGAMP/CpG‐loaded *μ*‐particles (*μ*GC) triggered a surge in the cytokine level (Figure 1c). Expression of IFN‐*β*‐regulated CCL‐5 followed the same pattern (Figure 1c). Although *μ*G treatment had no effect on TNF‐*α* expression and *μ*C only moderately stimulated cytokine expression, *μ*GC treatment drastically elevated TNF‐*α* level (Figure 1c). Enhancement of IFN‐*β* and TNF‐*α* expression was also observed in DCs treated with CpG/MPLA‐loaded *μ*‐particles (Figure S4, Supporting Information). In comparison, poly I:C‐loaded *μ*‐particles (*μ*poly I:C) did not promote strong IFN‐*β* expression in conventional DCs (cDCs) or pDCs (Figure S5, Supporting Information). Thus, loading soluble agonists into *μ*‐particle further enhanced cytokine expression and the response was receptor expression‐dependent, indicating synergy between pathways mediated by the respective agents in promoting cytokine production.

### 2.2 Characterization of the *μ*GC Particulate Adjuvant

We have previously shown that *μ*‐particles get trapped in endosomes for an extended amount of time and the process benefits both DC activation and antigen processing.^[^ ^25^ ^]^ In this study, we applied FITC‐labeled CpG to prepare fluorescent liposomal GC (LipoGC) and *μ*GC, applied them to treat immortalized DC2.4 dendritic cells, and monitored intracellular particle trafficking. Microscopic analysis detected cellular uptake of particles (Figure S6, Supporting Information), and colocalization of green fluorescent FITC‐CpG with early endosomes that were stained in red with an anti‐early endosome antigen 1 (EEA1) antibody in both treatments (Figure 1d). However, FITC‐CpG parted from early endosomes after 2 h of incubation in cells treated with LipoGC, while colocalization of FITC‐CpG and early endosomes could still be visualized 6 h after cells were treated with *μ*GC (Figure 1d). In addition, cells treated with *μ*GC displayed much stronger STING activation than those treated with LipoGC, as indicated by dramatically enhanced STING staining (Figure S7, Supporting Information). The result indicates sustained release of cGAMP and CpG into the endosomes in DCs treated with *μ*GC can lead to potent receptor activation.

In vivo particle uptake was monitored in live CD11c‐YFP transgenic mice. Cy5‐labeled *μ*GC particles were injected intradermally in the ear and time‐dependent particle uptake by fluorescent DCs was monitored under intravital microscopy. Overall, DCs displayed a gradient accumulation pattern around the *μ*GC particles (Figure 1e, upper panel), and Z‐stack analysis revealed cell‐associated particles either in the DC body or in spikes (Figure 1e, bottom panels) indicating cellular internalization of *μ*GC particles. In addition, most DCs in proximity of the particle inoculation site had developed spikes indicating their full maturation status, while those in the PBS‐treated ear showed less maturation‐related morphology (Figure 1f). Thus, *μ*GC inoculation attracts DC accumulation, resulting in effective particle uptake and DC maturation.

Transport of LipoGC and *μ*GC in the lymphatic system was tracked after the particles were inoculated into foot pads of TUBO tumor‐bearing mice. The TUBO tumor line was originally derived from mammary gland tumors developed in MMTV‐Her2 transgenic mice.^[^ ^31^ ^]^ Although both LipoGC and *μ*GC could be located in the sentinel popliteal lymph nodes 24 h after inoculation, most *μ*GC particles accumulated in the subcapsular space known for DC enrichment, while the LipoGC particles spread more evenly across the lymph node (Figure 1g, left panels). In addition, *μ*GC particles, but not LipoGC, could reach the inguinal lymph nodes in most mice (Figure 1g, right panels), indicating the *μ*GC particles were more effectively transported to the distant lymph nodes by DCs. DC maturation analysis corroborated particle biodistribution, as most DCs in the popliteal lymph nodes expressed maturation markers in both treatment groups, and more mature DCs were detected in inguinal lymph nodes in the *μ*GC treatment group (Figures S8 and S9, Supporting Information). We also applied small animal positron emission tomography‐computed tomography (PET‐CT) imaging to monitor time‐dependent particle transport at the whole‐body level. In primary TUBO tumor‐bearing BALB/c mice inoculated with ^64^Cu‐labeled particles in the footpads, *μ*GC particles were more efficiently transported to the lymph nodes, lymphatic organs, and the tumors than the empty *μ*‐particles (Figure 1h). Statistically significant elevation of *μ*GC particle accumulation in the popliteal lymph nodes and in the tumors was maintained 48 h after inoculation (Figure 1i). Higher accumulation in spleen was also detected at the 48 h time point (Figure S10, Supporting Information). These observations support effective uptake and transport of *μ*GC particles by DCs in the lymphatic system.

### 2.3 Antitumor Immune Responses from *μ*GCVax in Melanoma

**Figure 2:**
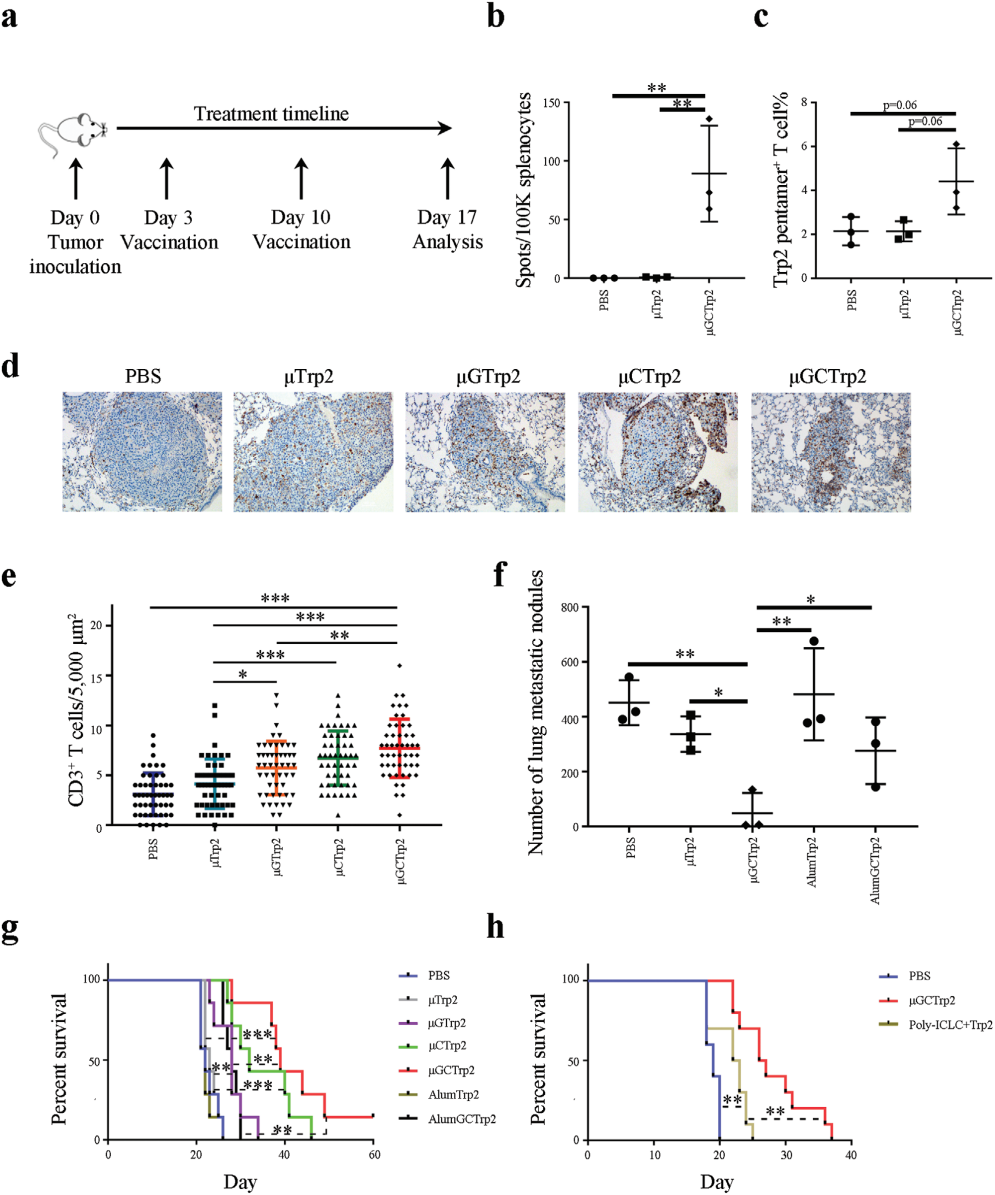
Antitumor activity from particulate vaccine in murine model of lung metastatic melanoma. a) Schematic view of treatment schedule. b) ELISpot assay of splenic T cells from mice treated with *μ*Trp2 and *μ*GCTrp2. *n* = 3 mice per group. Error bars, mean +/− SD. c) Flow cytometry analysis on Trp2‐specific T cell levels in the spleens of post‐vaccination mice. *n* = 3 mice per group. Error bars, mean +/− SD. d,e) Histological analysis on CD3^+^ T cell infiltration in metastatic B16 tumor nodules in post‐vaccination mice. Lung tissue blocks were stained with d) an anti‐CD3 antibody, and numbers of T cells inside the tumors were quantitated. e) Ten microscopic views per tissue block were analyzed. f) Evaluation of antitumor activity in metastatic B16 melanoma. Mice with metastatic B16 melanoma were treated twice with vaccines, and lung metastatic tumor nodules in different treatment groups were analyzed 2 weeks after the first treatment. *n* = 3 mice per group. Error bars, mean +/− SD. g) Evaluation of therapeutic efficacy from different treatment groups. Kaplan–Meier plots were generated based on animal survival in different vaccination groups. *n* = 7 mice per group. h) Comparison on therapeutic efficacy from *μ*GCTrp2 and poly‐ICLC‐based Trp2 peptide vaccine. Kaplan–Meier plots were generated based on animal survival in different vaccination groups. *n* = 10 mice per group. Statistics: One‐way ANOVA for multi‐group comparison. *: *p* < 0.05; **: *p* < 0.01; ***: *p* < 0.001.

Based on the above characterization, we reasoned that *μ*GC could serve as an ideal adjuvant for cancer vaccine (*μ*GCVax) development. To test this hypothesis, we packaged *μ*GC with melanoma Trp2^180‐188^, an antigenic peptide shared by murine H‐2K^b^ and human HLA‐A*0201 major histocompatibility complex (MHC) proteins,^[^ ^32^ ^]^ and applied the vaccine (*μ*GCTrp2) to treat C57BL/6 mice bearing lung metastatic B16 melanoma (**Figure** **2**a). We detected high numbers of IFN‐*γ*‐producing T cells (Figure 2b and Figure S11, Supporting Information) and antigen‐specific T cells (Figure 2c) in splenocytes from mice treated with *μ*GCTrp2, but not with *μ*Trp2. Splenocytes isolated from *μ*GCTrp2‐treated mice also produced high levels of CCL5 and IFN‐*γ* in an ex vivo setting (Figure S12, Supporting Information). Histological analysis revealed significantly increased levels of tumor‐infiltrated T cells after treatment with *μ*GTrp2, *μ*CTrp2, or *μ*GCTrp2 (Figure 2d,e); however, T cells in the *μ*CTrp2 treatment group were concentrated at the tumor periphery while those in the *μ*GTrp2 and *μ*GCTrp2 treatment groups penetrated more evenly across the tumor nodules (Figure 2d). In line with the observation, flow cytometry analysis detected a surge in CD8^+^ T cells after *μ*GCTrp2 treatment in peripheral blood, lymph nodes, spleens, and tumor‐bearing lungs (Figure S13, Supporting Information). In addition, *μ*GCTrp2 treatment also raised levels of T memory cells in the lymph nodes and tumor‐bearing lungs (Figure S14, Supporting Information). Interestingly, less than 20% CD8^+^ T cells in the tumor‐bearing lungs expressed PD‐1, although the number was higher than CD8^+^ T cells in the lymph nodes (Figure S15, Supporting Information). These results indicate that *μ*GCVax treatment activates T cells and triggers tumor infiltration of these cells.

Tumor infiltration of T cells and antitumor efficacy were evaluated in mice with lung metastatic B16 melanoma. Treatment with *μ*GTrp2, *μ*CTrp2, or *μ*GCTrp2 significantly enhanced T cell infiltration into the tumor nodules (Figure 2e), and dramatic reduction in number of tumor nodules in the lung was observed in the *μ*GCTrp2 treatment group (Figure 2f). The antitumor activity was *μ*‐particle‐dependent, as replacing *μ*‐particles with alum adjuvant, a commonly used vaccine adjuvant with a comparable size range that activates the NALP3 inflammasome,^[^ ^23^, ^33^ ^]^ abolished antitumor activity (Figure 2f,g). In addition, adjuvant activity from *μ*GC was compared with poly‐ICLC consisting of polyinosinic and polycytidylic acid stabilized with poly‐L‐lysine and carboxymethylcellulose, an adjuvant that has been applied in recent clinical trials with neoantigen‐based peptide vaccines for human cancer treatment.^[^ ^34^, ^35^ ^]^ Side‐by‐side comparison showed significantly improved survival benefit from *μ*GCTrp2 over poly‐ICLC‐based Trp2 peptide vaccine (Figure 2h).

### 2.4 Antitumor Immunity from *μ*GCVax in Breast and Colorectal Cancers

**Figure 3:**
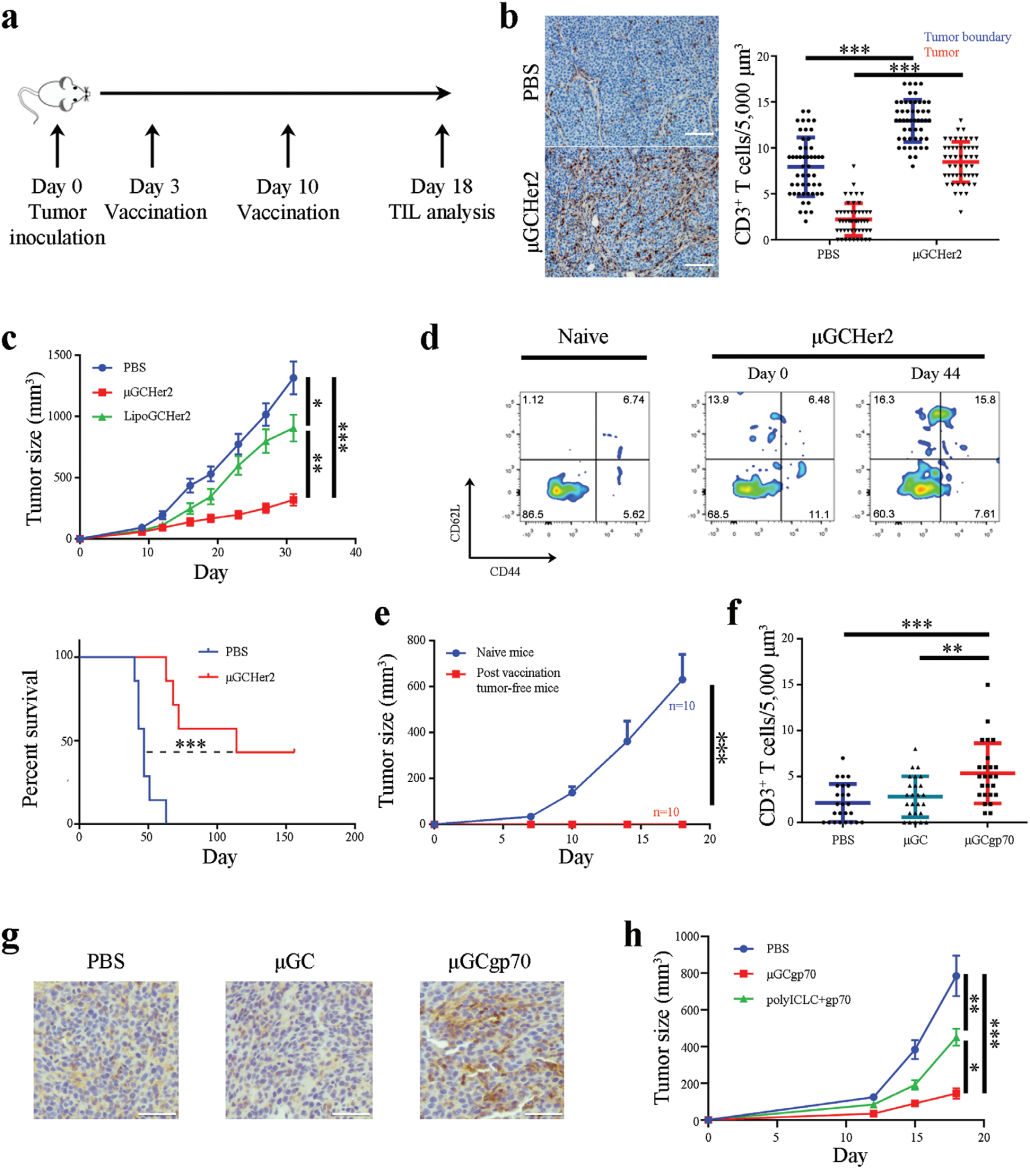
Antitumor immunity from particulate vaccines in murine models of Her2‐positive breast cancer and colorectal cancer. a) Schematic view of treatment schedule for TUBO tumor‐bearing mice. b) Analysis on CD3^+^ T cell infiltration in TUBO tumors after mice were treated with *μ*GCHer2. *n* = 3 mice per group. TUBO tumor tissue blocks were stained with an anti‐CD3 antibody (in brown), and number of T cells inside the tumors were quantitated. Ten microscopic views per tissue block were analyzed. T cell density was determined both in the tumor boundary and inside the tumor parenchyma. Bar: 100 µm. c) Therapeutic efficacy evaluation in BALB/c mice with primary TUBO tumors (*n* = 7 mice per group). Kaplan–Meier plot was generated based on animal survival. *n* = 7 mice per group. d) Flow cytometry analysis of memory T cells in post *μ*GCHer2 vaccination tumor‐free mice. e) Therapeutic efficacy evaluation in naïve mice and post‐vaccination tumor‐free mice after they were inoculated with TUBO tumor cells in the mammary gland fat pads. *n* = 10 mice per group. Error bars: mean +/‐ SEM. f) Analysis on CD3^+^ T cell infiltration in CT26 tumors after mice were treated with *μ*GCgp70. *n* = 3 mice per group. Number of CD3^+^ T cells were counted in ten microscopic views and analyzed. g) Representative images of CT26 tumor tissue blocks stained with an anti‐CD3 antibody (in brown). Bar: 50 µm. h) Comparison on therapeutic efficacy from *μ*GCgp70 and polyICLC‐based gp70 peptide vaccine based on tumor growth. *n* = 10 mice per group. Error bars: mean +/− SEM. Statistics: One‐way ANOVA for multi‐group comparison, Unpaired Student's *t*‐test for comparison between two groups and log‐rank tests for Kaplan–Meier plots. *: *p* < 0.05; **: *p* < 0.01; ***: *p* < 0.001.

Breast cancer is generally considered as noninflamed, and patients with breast cancer have responded poorly to therapy with checkpoint blockade antibodies.^[^ ^36^, ^37^ ^]^ To test whether a *μ*GCVax could also stimulate antitumor immune response in breast cancer, we prepared a breast cancer‐specific vaccine by replacing Trp2 peptide with Her2p66 (*μ*GCHer2) which is a class I Her2 peptide.^[^ ^25^, ^38^ ^]^ BALB/c mice with primary Her2‐positive TUBO tumors were treated with *μ*GCHer2, and tumor infiltration of T cells was analyzed (**Figure** **3**a). The multicentric structure of TUBO tumor nodules allowed us to quantitatively analyze T cell numbers in different areas of the tumor bed (Figure S16, Supporting Information). As expected, T cells were sparse in tumors from untreated control mice, with less T cells in the center of tumor nodules (2 cells/5,000 µm^3^) than at the boundary region (7.8 cells/5,000 µm^3^). Upon vaccination, T cell density increased across the whole tumor, ranging from 8.5 cells/5,000 µm^3^ in the center to 13 cells/5,000 µm^3^ at the tumor boundary (Figure 3b). Vaccination caused a significant delay in tumor growth (Figure 3c), and 40% of the mice in the treatment group achieved tumor remission (Figure S17, Supporting Information). Single cell analysis of post‐vaccination tumor samples revealed dramatically reduced levels of CD11b^+^Ly6C^int^Ly6G^+^ polymorphonuclear cells (PMN cells, also known as granulocytes) (Figure S18, Supporting Information). This population of myeloid cells is known to cause resistance to immunotherapy.^[^ ^39^ ^]^ Eliminating PMN cells from the tumor tissue may sensitize treatment. Analysis of peripheral blood samples from the tumor‐free mice revealed elevated levels of both CD44^high^CD62L^low^ effector memory T cells and CD44^high^CD62L^high^ central memory T cells (Figure 3d). Memory T cells are known to provide long‐term protection against tumors carrying matched antigens.^[^ ^40^ ^]^ As expected, no tumor formation was detected in any of the post‐vaccination tumor‐free mice when they were re‐challenged with TUBO tumor cell inoculation in the memory gland fat pads (Figure 3e).

Colorectal cancer is another cancer type that is generally considered as noninflamed, and only a small fraction of colorectal cancers respond favorably to checkpoint blockade antibody treatment.^[^ ^41^ ^]^ Since murine CT26 colorectal tumor cells were known to express a gp70 tumor‐associated antigen,^[^ ^42^ ^]^ we applied a gp70‐specific class I antigen peptide to prepare therapeutic vaccine (*μ*GCgp70) and used it to treat BALB/c mice carrying CT26 tumors. Immunohistochemical staining revealed significantly increased levels of tumor‐infiltrated CD3^±^ T cells in the *μ*GCgp70 treatment group over size‐matched tumors from phosphate buffered saline (PBS) control group or those treated with adjuvant only (Figure 3f,g). As a result, *μ*GCgp70 treatment dramatically inhibited CT26 tumor growth (Figure 3h). In addition, tumor inhibitory effect from *μ*GCgp70 was superior over a polyICLC‐based vaccine carrying the same antigen peptide (Figure 3h).

Taken together, *μ*GCVax is effective in stimulating sustained antitumor immune responses and can provide potent antitumor immunity in the noninflamed breast and colorectal cancers.

### 2.5 Contribution from DC Subpopulations on Antitumor Immunity

**Figure 4:**
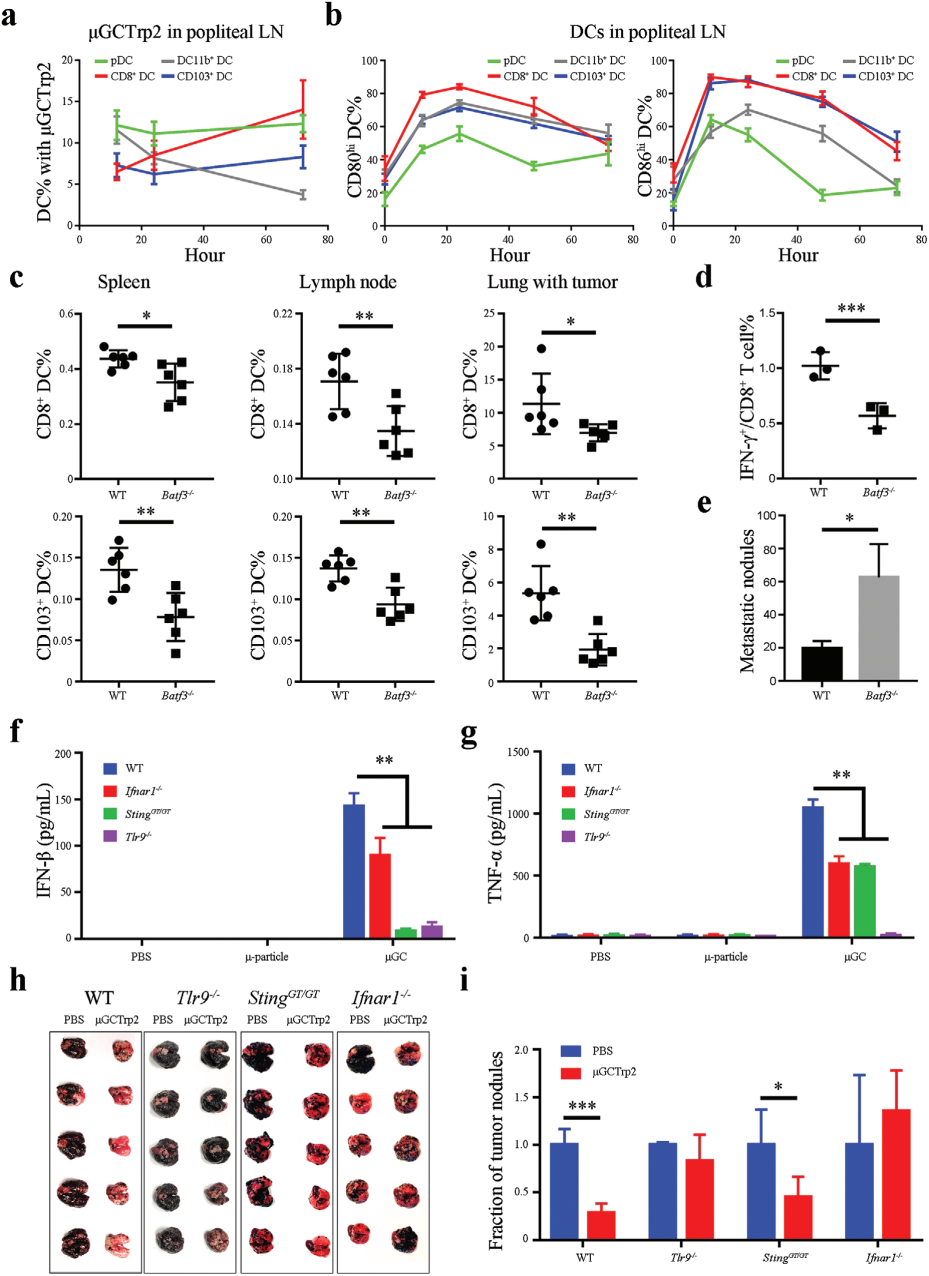
DC subpopulations and key genes in mediating vaccine particle transport and antitumor immunity. a) Time‐dependent transport of FITC‐labeled *μ*GCTrp2 vaccine to popliteal lymph nodes by different subpopulations of DCs. Popliteal lymph nodes were collected from B16 tumor‐bearing mice 12, 24 or 72 h after they were treated with FITC‐labeled *μ*GCTrp2 in the foot pads, and flow cytometry was applied to analyze percentages of FITC‐positive CD8^+^ DC, CD11b^+^ DC, CD103^+^ DC, and pDC. *n* = 3 mice per group. Error bars: mean +/− SD. b) Time‐dependent changes of maturation markers in DCs from popliteal lymph nodes. c) Flow cytometry analysis on levels of CD8^+^ DCs and CD103^+^ DCs in spleens, lymph nodes, and tumor‐bearing lungs from wild‐type (WT) and *Batf3* ^−/−^ mice. *n* = 6 mice per group. Error bars: mean +/− SD. d) Flow cytometry analysis on levels of activated CD8^+^ T cells in popliteal lymph nodes from WT and *Batf3* ^−/−^ mice treated with *μ*GCTrp2 vaccine. *n* = 3 mice per group. Error bars: mean +/− SD. e) Quantitative analysis on metastatic tumor nodules in the lungs from WT and *Batf3* ^−/−^ mice treated with *μ*GCTrp2. *n* = 12 mice per group. Error bars: mean +/− SEM. f,g) Stimulation of cytokine expression in WT and mutant BMDCs. BMDCs from WT, *Ifnar1* ^−/−^, *Sting* ^GT/GT^, and *Tlr9* ^−/−^ mice were treated with *μ*‐particle or *μ*GC for 24 h, and levels of f) IFN‐*β* and g) TNF‐*α* in growth media were measured and compared. *n* = 3 mice per group. Error bars: mean +/− SD. h,i) Antitumor activity in WT and mutant mice. WT and knockout mice bearing lung metastatic B16 tumors were treated twice with PBS control or *μ*GCTrp2. Mice were euthanized on day 17, and lungs were collected and number of B16 tumor nodules was quantitated. Statistics: ANOVA for multi‐group comparison and Student's *t‐*test for comparison between two groups. *: *p* < 0.05; **: *p* < 0.01; ***: *p* < 0.001.

DCs are a mixed population of antigen‐presenting cells with diverse and sometimes opposing functions.^[^ ^43^ ^]^ In order to identify DC subpopulations responsible for vaccine particle transport and subsequent antigen presentation, we treated tumor‐bearing C57BL/6 mice with FITC‐labeled *μ*GCTrp2 and tracked vaccine particle distribution in DCs in the draining lymph nodes. Flow cytometry was applied to quantitate FITC‐positive cDCs (CD8^+^ DCs, CD11b^+^ DCs, and CD103^+^ DCs) and B220^+^ pDCs (Figure S19, Supporting Information). The analysis revealed persistent accumulation of vaccine particles in CD8^+^ DCs, sustained levels of pDCs and CD103^+^ DCs with particles, and steady reduction of particle‐associated CD11b^+^ DCs (**Figure** **4**a). In addition, most cDCs in the draining lymph node kept an activation status based on CD80 and CD86 expression levels for up to 72 h after vaccination, while pDCs lost CD86 expression quickly (Figure 4b). Different DC subpopulations were most likely mobilized to the inoculation site as a result of modification of the local microenvironment after *μ*GCTrp2 treatment, as demonstrated by migration of the Langerhans cells from the epidermis layer to the dermis layer at the vaccination site (Figure S20, Supporting Information). The result indicates that the cDCs and pDCs play a major role on transport of vaccine particles into lymph nodes.

cDCs are known to mediate Th1 T cell immunity,^[^ ^11^, ^44^, ^45^, ^46^ ^]^ and the *Batf3* gene is essential for CD8^+^ and CD103^+^ DC maturation.^[^ ^47^, ^48^ ^]^ We treated wild‐type (WT) and *Batf3^−/−^* knockout mice carrying lung metastatic B16 tumors with *μ*GCTrp2 to evaluate antitumor immunity. As expected, *Batf3^−/−^* mice had significantly reduced levels of CD8^+^ and CD103^+^ DCs in the spleens and popliteal lymph nodes compared to wild‐type mice (Figure 4c). There were also fewer tumor‐associated CD8^+^ and CD103^+^ DCs in the *Batf3* ^−/−^ mice than in wild‐type mice (Figure 4c). We applied Trp2 antigen peptides to challenge equal number of splenic CD8^+^ T cells from post vaccination mice, and detected significantly less number of IFN‐*γ*‐producing cells from *Batf3* ^−/−^ mice than from wild‐type mice (Figure 4d). Consistent with the finding, *Batf3* ^−/−^ mice had diminished ability to combat lung metastatic tumors compared to wild‐type mice upon *μ*GCTrp2 treatment (Figure 4e). In a separate study, we treated mice with an anti‐PDCA1 antibody to assess the impact from pDCs on tumor growth. Since PDCA1 (CD317) is specifically expressed on murine pDCs, treatment with anti‐PDCA1 antibody can effectively deplete pDCs.^[^ ^49^ ^]^ Surprisingly, the treatment did not abrogate inhibitory effect from the vaccine (Figure S21, Supporting Information). Based on these observations, we concluded that the CD8^+^ and CD103^+^ cDCs play a major role in mediating *μ*GCVax activity.

### 2.6 Contribution from IFN‐I Signaling on Antitumor Immunity

Pathways critical for *μ*GCVax activity were examined in *Sting^GT/GT^*, *Tlr9^−/−^*, and *Ifnar1^−/−^* mice.^[^ ^50^, ^51^ ^]^ The *Sting^GT/GT^* mice carry a point mutation that functions as a null allele.^[^ ^52^ ^]^ In an in vitro setting, GM‐CSF/IL4‐induced BMDCs derived from wild‐type, *Sting^GT/GT^*, *Tlr9^−/−^*, and *Ifnar1^−/−^* mice were treated with *μ*GC particles, and stimulation of IFN‐*β* and TNF‐*α* secretion was determined. As expected, *μ*GC potently stimulated IFN‐*β* and TNF‐*α* expression in BMDCs derived from WT mice, but the stimulatory activity was partially lost in *Ifnar1^−/−^* BMDCs (Figure 4f,g). Stimulation of IFN‐*α*/*β* expression was also lost in pDCs derived from *Ifnar1^−/−^* (Figure S22, Supporting Information). The results were in line with a previous report and inhibition of IFN‐*β* expression was most likely due to loss of an IFN‐dependent amplification loop.^[^ ^51^ ^]^ While BMDCs derived from *Tlr9^−/−^* did not express IFN‐*β* or TNF‐*α*, those from *Sting^GT/GT^* still expressed TNF‐*a*, but not IFN‐*β*, in response to *μ*GC challenge (Figure 4f,g).

Antitumor activity was compared in WT and knockout mice carrying lung metastatic B16 tumors. In line with the efficacy study from *μ*GCTrp2 (Figure 2f), vaccination reduced total number of tumor nodules by over 70% in the lungs of WT mice, and the remaining tumor nodules were much smaller than those in the PBS control group (Figure 4h,i). Deletion of the *Tlr9* or *Ifnar1* gene rendered the vaccine ineffective in blocking tumor formation. Interestingly, partial antitumor activity from *μ*GCTrp2 retained in *Sting^GT/GT^* mice (Figure 4h,i). These results indicate that TLR9 and IFN‐I receptor‐mediated pathways are essential vaccine activity, while the STING signaling may play a supporting role in mediating antitumor immunity against metastatic B16 tumor.

## 3 Discussion and Conclusion

The TLR and STING pathways play important roles on host defense. Combination of different ligands/agonists can have either a synergistic or an antagonistic effect depending on the test model, such as synergy between TLR3 and TLR9 ligands on monocytes and opposite roles between TLR and STING signaling on macrophages.^[^ ^53^, ^54^ ^]^ Our screening identified synergy between selected ligands on stimulation of IFN‐*β* and TNF‐*α* expression in BMDCs. In addition, we demonstrated that loading soluble TLR ligands and STING agonist into the *μ*‐particles further enhanced cytokine expression, potentially as a result of crosstalk between the TLR and STING‐mediated pathways and *μ*‐particle‐activated TRIF/MAVS signaling.^[^ ^25^ ^]^

Although it has been previously shown that lipid nanoparticles can mediate delivery of therapeutic agents to lymph nodes and other lymphatic organs,^[^ ^55^, ^56^ ^]^ our result indicated that *μ*GC particles could be more effectively taken up and transported to the lymph nodes by DCs than the smaller LipoGC particles based on their different distribution pattern in the sentinel popliteal lymph nodes and more distant inguinal lymph nodes. Another interesting observation was that more GC‐loaded *μ*‐particles accumulated in the tumor tissue than empty *μ*‐particles. It has been well documented that tumors contain tertiary lymphoid structures which play important roles in mediating adaptive antitumor immune responses.^[^ ^57^ ^]^ Indeed, such structures enable tumor infiltration of both naïve T cells and memory T cells, and produce different set of cytokines from the rest tumor tissue.^[^ ^58^ ^]^ These observations, together with sustained endosomal retention and potent DC activation, provided strong support for *μ*GC as a particulate adjuvant in cancer vaccine development.

The finding that cDCs played a predominant role in mediating vaccine activity in the selected murine tumor models is intriguing, since both cDCs and pDCs were involved in vaccine particle transport to draining lymph nodes. In addition, although many studies have demonstrated that the *Batf3*‐dependent cDCs, especially CD103^+^ DCs, are essential for priming of tumor‐specific CD8^+^ T cells,^[^ ^44^, ^45^, ^59^ ^]^ there have also been reports on successful application of pDC‐based cancer vaccines.^[^ ^60^, ^61^ ^]^ Interestingly, a recent study revealed that cross‐presenting pDCs required cDCs to achieve cross‐priming in vivo.^[^ ^62^ ^]^ In a separate study, it was demonstrated that peptide DC vaccines prepared with cDCs generated more robust Th1 type immune responses than those with pDCs.^[^ ^63^ ^]^ Another surprise finding is higher potency from the *μ*GCVax than a polyICLC‐based vaccine. In line with the study, it was previous found that a nanoparticle‐based vaccine carrying a STING‐activating polymer provided better therapeutic outcome than a polyIC‐based vaccine. It is unknown whether low TLR3 expression in murine DCs is responsible for poor efficacy from the polyIC‐based vaccines.

Therapeutic vaccines have shown promising therapeutic efficacy in inflamed cancer types such as melanoma.^[^ ^34^, ^64^, ^65^ ^]^ Recent progress in clinic has also demonstrated their applicability in glioblastoma.^[^ ^35^, ^66^ ^]^ With the advancement in genome sequencing to detect gene mutations and in neoantigen prediction,^[^ ^67^, ^68^, ^69^, ^70^ ^]^ there will be growing demand for personalized vaccination based on the mutation spectrum in individual patients. Thus, it is critical to ensure that the personalized vaccines can function in both in inflamed and noninflamed cancer types. Results from the current study fully support application of *μ*GCVax for the exact purpose, fulfilling the goal of personalized cancer immunotherapy.

In conclusion, we have developed a particulate vaccine platform to potentiate antitumor immunity. The *μ*GCVax is effective in mounting antitumor immune responses in multiple tumor types including both inflamed and noninflamed tumors. This platform offers a vital tool for the development of personalized cancer treatment.

## 4 Experimental Section

##### Mouse Strains

All mice were housed at the Houston Methodist Research Institute (HMRI) vivarium and were maintained under pathogen‐free conditions in accordance with regulatory standards of the National Institute of Health and American Association of Laboratory Animal Care standards. All procedures were approved by the HMRI Institution of Animal Care and Use Committee (accreditation number AUP06200042). BALB/c and C57BL/6 mice were purchased from Charles River Laboratories. The *Batf3^−/−^*, *Ifnar1^−/−^*, *Sting^GT/GT^*, and CD11c‐EYFP mice were purchased from the Jackson Laboratory, and *Tlr9^−/−^* mice were originally from the Akira laboratory. All genetically engineered mice were maintained in HMRI.

##### Antibodies, Adjuvants ELISA Kits, Antigen Peptides, and Pentamer

The following antibodies were used in this study: B220 clone RA3‐682, CD8*α* clone 53–6.7, CD11b clone M1/70, CD40 clone 3/23, CD80 clone 16‐10A1, LY‐6C clone AL‐21, MHCII clone M5/114.15.2, APC‐Cy7 hamster anti‐mouse CD3e from BD‐Bioscience. CD64 clone X54‐5/7.1, PE‐anti‐mouse/human CD207 (Langerin) Ab clone 4C7, Ly6C clone HK1.4, PD‐1 clone 29F.1A12, APC‐anti‐TLR3 antibody clone 11F8, APC‐anti‐TLR4 antibody clone SA15‐21, PE‐anti‐TLR7 antibody clone A94B10, and PE‐anti‐TLR‐9 antibody clone S18025A from Biolegend. Anti‐STING polyclonal antibody was from ProteinTech. Rat anti‐PDCA1 antibody and control IgG were from BioXCell. CD4 clone GK1.5, CD62L clone MEL‐14, CD103 clone 2E7, and anti‐mouse IFN‐*γ* clone R4‐6A2 from eBiosciences. Pacific Blue conjugated CD45 rat anti‐mouse mAb, PE‐labeled CD62L (L‐Selectin) mAb from Thermo Fisher, CD3e clone 145‐2C11, CD11c clone N418, CD86 clone GL‐1, APC‐anti‐mouse CD4 clone RM4‐5, PerCP‐Cy5.5 anti‐mouse CD8*α* clone 2.43, FITC‐anti‐human/mouse CD44 clone IM7 from Tonbo Biosciences. The following Toll‐like receptor (TLR) ligands and STING agonist were applied to stimulate cytokine and chemokine secretion in BMDCs and to prepare vaccine particles: CpG oligonucleotide ODN 1826 class B CpG oligonucleotide (CpG), 2′3′‐cGAMP (cGAMP), monophosphoryl lipid A (MPLA), and polyinosine‐polycytidylic acid (polyI:C) from Invivogen, R848 from Sigma Aldrich. Flt3 ligand was purchased from Tonbo. These ELISA kits were used to determine cytokine and chemokine levels in cell growth media: ELISA kits for TNF‐*α* and IFN‐*α* from Invitrogen, ELISA kit for CCL‐5 from R&D Systems, and ELISA kit for IFN‐*β* from PBL Assay Science. The Her2‐specific p66 antigen peptide TYVPANASL, Trp2 antigen peptide SVYDFFVWL, and gp70 antigen peptide SPSYVYHQF were purchased from GenScript. Trp2 pentamer was from Immudex.

##### Generation and In Vitro Stimulation of BMDCs

BMDCs were generated by induction with GM‐CSF/IL‐4 or Flt3 ligand. To generate GM‐CSF/IL‐4‐induced BMDCS, bone marrow cells were flushed out from femur and tibia with 2% fetal bovine serum‐containing PBS. After removal of red blood cells, bone marrow cells were grown in a 37 °C incubator with 5% CO_2_ in RPMI‐1640 supplemented with 20 ng mL^−1^ recombinant murine GM‐CSF and IL‐4 for 6 days. Cell culture medium was refreshed every other day. To induce BMDC with Flt3 ligand, bone marrow cell culture was supplemented with 200 ng mL^−1^ Flt3 ligand. Cell culture medium was refreshed on day 5, and continued for another 5 days. To test stimulation of GM‐CSF/IL‐4‐induced BMDC, immature BMDCs were suspended in RPMI‐1640 medium without GM‐CSF or IL‐4, and seeded into 24‐well plates at a seeding density of 0.5 × 10^6^ cells per well. Cells were treated with partial or complete liposomal vaccines or *μ*‐vaccines. After treatment for 24 h, cell growth media were collected for ELISA analysis to measure expression levels of IFN‐*α*/*β*, IL‐12p70, CCL5, and TNF‐*α*, and cells were stained with anti‐CD40, anti‐CD80, or anti‐CD86 antibody to determine maturation status by flow cytometry. CD8^+^ DCs and B220^+^ pDCs were isolated from Flt3L‐induced BMDCs with a CD8^+^ DC isolation kit (Miltenyi) or B220 microbeads (Miltenyi) before they were applied for stimulation assay.

##### Stimulation of Cytokine Production by TLR Ligands and STING Agonist

BMDCs were seeded into 24‐well plates at a seeding density of 5 × 10^5^ cells per well, and treated with the following reagents either as a single agent or in combination: 2.5 µg mL^−1^ CpG, 1.25 µg mL^−1^ cGAMP, 0.5 µg mL^−1^ MPLA, 0.5 µg mL^−1^ polyI:C, 0.5 µg mL^−1^ R848. Cell growth media were collected 2 and 24 h later, and levels of IFN‐*β*, CCL‐5, and TNF‐*α* were measured with ELISA kits.

##### Preparation of Particulate Vaccine and Poly‐ICLC Vaccine

The *μ*‐particles were produced and chemically modified as we have previously described.^[^ ^30^ ^]^ Liposomes encapsulated with soluble adjuvants and antigens were prepared as described.^[^ ^25^ ^]^ Briefly, soluble adjuvants and antigens were dissolved in water, and mixed with 20 mg mL^−1^ 1,2‐dioleoyl‐*sn*‐glycero‐3‐phosphocholine, *t*‐butanol, and 0.1% Tween‐20. The sample was then freeze‐dried in a lyophilizer. Liposomes were reconstituted by adding water into the powder, and were then loaded into *μ*‐particles by brief sonication. The complete *μ*GCVax contains 10 µg CpG, 5 µg cGAMP, and 100 µg antigen peptide (the p66 antigen peptide, gp70 peptide, or Trp2 antigen peptide) in 0.6 billion 1 µm particles. The vaccine particles were resuspended in 50 µL water. To measure loading efficiency of μGCVax, 10% CpG was replaced by Cy5‐labeled CpG to prepare fluorescent vaccine particle. After loading, μGCVax particles were spun down at 21 000 x *g* for 10 min. Supernatant was collected, diluted 1:20 with H_2_O, and fluorescent intensity was measured at Ex 630 nm/Em 671 nm with a BioTek Synergy H4 microplate reader. High‐performance liquid chromatography (HPLC) was also applied to measure reagent content in μGCVax. Briefly, 2′3′‐cGAMP, CpG oligomer, and antigen peptide were separated by reverse phase chromatography in an Agilent Technologies 1260 Infinity II HPLC system with a ZORBAX 300SB‐C18 column (4.6 × 150 mm, 3.5 µm particle size) (Santa Clara, CA, USA). The mobile phases were acetonitrile (phase A) and 0.1% trifluoroacetic acid in H_2_O (phase B). The mobile phase started at 1% A and 99% B for 4 min, and was then changed to 90% A and 10% B in a linear manner between 4 and 19 min. Absorption peaks were detected with a diode array detector at 254 nm wavelength. Particles with over 95% loading capacity passed quality control. To prepare poly‐ICLC vaccine with Trp2 peptide, 50 µg poly‐ICLC is mixed with 100 µg Trp2 peptide in a final volume of 50 µL aqueous solution.

##### Intravital Microscopic Analysis of Particle Uptake

Liposomal CpG and cGAMP were loaded into Cy5‐labeled *μ*‐particles, and each CD11c‐EYFP mouse was injected subcutaneously in the left ear with 5 × 10^7^ *μ*GC. Mice were placed under a Nikon A1 two‐photon intravital microscope and migration of EYFP‐expressing DCs and their capture of Cy5‐positive *μ*GC particles were recorded on daily basis in the next 5 days.

##### Vaccine Particle Transport

Transport of vaccine particles was monitored by tracking FITC‐labeled vaccines in popliteal and inguinal lymph nodes and ^64^Cu‐labeled vaccine particles across the whole body. To monitor vaccine particle transport in lymph nodes, LipoGC and *μ*GC vaccine particles prepared with FITC‐CpG were inoculated into the foot pads of female BALB/c mice bearing primary TUBO tumors. Mice were euthanized 24 h later, and popliteal and inguinal lymph nodes were harvested. Tissue sections were processed for staining with 4′,6‐diamidino‐2‐phenylindole (DAPI) and anti‐CD11c antibody, and fluorescent microscopic images were captured. To monitor vaccine particle transport across the whole body, *μ*‐particles were conjugated with 1,4,7,10‐tetraazacyclododecane‐1,4,7,10‐tetraacetic acid mono‐N‐hydroxysuccinimide (DOTA‐NHS) ester, and incubated with ^64^Cu before they were loaded with liposomal CpG and cGAMP. Mice bearing primary TUBO tumors were inoculated in the foot pads with either ^64^Cu‐labeled empty *μ*‐particles or ^64^Cu‐labeled *μ*GC particles, and radiation intensity across the whole body was monitored with PET‐CT at the 3, 6, 10, 24, and 48 h time points. Mice were euthanized 48 h after particle inoculation, and biodistribution of the radioactive vaccine particles in major organs, lymph nodes, and the tumor was determined.

##### Flow Cytometry Analysis

Flow cytometry was applied to determine TLR and STING expression and sub‐population of DCs (CD8^+^ DC, CD11b^+^ DC, CD103^+^ DC, and pDC) in popliteal lymph nodes and spleens following a recently described approach.^[^ ^45^ ^]^ Cells were treated with a BD Cytofix/Cytoperm fixation/permeablization kit before antibody incubation. Flow cytometry was also applied to analyze total CD4^+^ T cells (CD3^+^CD4^+^), total CD8^+^ T cells (CD3^+^CD8^+^), antigen‐specific T cells (CD3^+^CD8^+^pentamer^+^ or CD3^+^CD8^+^IFN‐*γ* ^+^), and memory T cells (CD44^+^CD62L^+^ central memory T cells and CD44^+^CD62L^−^ effect memory T cells) in peripheral blood, spleen, and tumor tissues.

##### T Cell Activation Assays

To study T cell activation ex vivo, C57BL6 mice were inoculated with B16 melanoma (on day 0), and treated twice (on days 3 and 10) with partial or complete vaccines containing 100 µg Trp2 peptide/mouse in the foot pads. Mice were euthanized 7 days after the second vaccination (on day 17), and spleens were collected to process for single cell isolation. ELISpot assay was applied to determine antigen‐specific T cell activity. Briefly, splenocytes were seeded at 1 × 10^5^ cell per well in an anti‐IFN‐*γ*‐coated MultiScreen‐IP plate (Millipore), and stimulated with 10 µg mL^−1^ Trp2 peptides in growth medium for 36 h. The plate was then washed and incubated with biotinylated anti‐mouse IFN‐*γ* antibody, followed by staining with an avidin‐HRP (Ebioscience). In a separate study, splenocytes were seeded into 24‐well plates at a seeding density of 1 × 10^5^ cell per well and treated with 10 µg mL^−1^ Trp2 peptide. Cell culture medium was collected 24 h later, and CCL5 and IFN‐*γ* levels in growth media were determined with ELISA. CD8^+^ T cells were also analyzed with Trp2 pentamer.

##### Evaluation of Antitumor Efficacy in Murine Tumor Models

Murine model of lung metastatic melanoma was generated by inoculating murine B16 melanoma cells at 2.5 × 10^5^ cells per mouse by tail vein injection into 6 to 8 weeks old C57BL6 mice. Three days after tumor inoculation, mice were randomly allocated into treatment groups, and treated with partial or complete vaccines. They were boosted with vaccines one more time 1 week after the first vaccine. Mice were euthanized 15 days after the first vaccination, and number of black metastatic tumor nodules in the lung was counted. The same treatment procedure was applied to compare lung metastatic B16 tumor formation in wild‐type and gene knockout mice (*Sting^GT/GT^*, *Tlr9^−/−^*, and *Ifnar1^−/−^*) in the C57BL6 background. In the survival study, mice went through the same treatments, and were euthanized when they reached one of the endpoints including lethargic, hunched back, ruffled fur, loss of 15% body weight, and difficulty in breathing. To test therapeutic efficacy from Her2‐specific vaccines, BALB/c mice were inoculated with Her2‐expressing TUBO tumor cells in the mammary gland fat pads at 1 × 10^6^ cells per mouse. They were inoculated with *μ*GCVax in the fat pads once 3 days after tumor inoculation and the second time 1 week after the first vaccination. Tumor growth was monitored on daily basis. Mice were euthanized when diameter of the tumor exceeds 2 cm or one of the endpoints is met including tumor ulceration, lethargic, hunched back, ruffled fur, and loss of 15% body weight. The same endpoints applied to mice with CT26 tumors. To challenge mice that were tumor‐free after vaccination, mice were inoculated with 1 × 10^6^ TUBO cells per mouse in the fat pad, and tumor growth was monitored in the next 20 days. To test therapeutic efficacy after pDC depletion, tumor‐bearing mice were treated with 250 µg anti‐PDCA1 antibody or control IgG intraperitoneally 1 day and 3 days before vaccination and every other day after vaccination for 14 days. Mice were inoculated with *μ*GCTrp2 on day 4 and day 11 post tumor inoculation, and tumor growth and animal survival were monitored.

##### Statistical Analysis

Statistical analyses were performed with the GraphPad Prism 5 (GraphPad Software, Inc., CA, USA) and SAS 9.4 (SAS institute Inc., NC, USA). For all in vivo analysis, sample sizes were chosen to ensure adequate power to detect a pre‐specified effect size. Animals were randomized prior to treatment. Blinding was not performed. Differences were evaluated by *F*‐tests of the fixed effects of a two‐way analysis of variance (ANOVA) model which allowed for the heterogenous nature of the data and included a random subject effect to account for the data's correlation structure. Dunnett's or Tukey's adjustment was applied for multiple comparisons to correct for multiplicity. Survival was estimated by the Kaplan–Meier method and differences in survival were evaluated using log‐rank tests. Global tests were done to establish that significant differences exist, and then pairwise group comparisons were made and adjusted for multiplicity using Hommel's approach. *p*‐Values of less than 0.05 and 0.01 were considered statistically significant and very significant, respectively. Data were presented as means ± SD or SEM.

## Conflict of Interest

The authors declare no conflict of interest.

## Author Contribution

H.S. developed the concept and supervised experiments. H.S., J.M., X.X., E.A.M., and M.F. prepared the manuscript. X.L. provided silicon microparticles, and Z.L., J.Z., J.L., H.L., J.S., J.M., M.R, F.L. K.Y., and Z.L. prepared reagents and carried out in vitro and in vivo biological studies. J.M. and Z.L. performed statistical analysis.

## Supporting information

Supporting Information

## Data Availability

All data associated with this study are presented in the manuscript. Reagents used in this study are readily available and will be provided under the material transfer policies of Houston Methodist Research Institute.
