# Correction to “Synergistic Activation of Antitumor Immunity by a Particulate Therapeutic Vaccine”

**DOI:** 10.1002/advs.202414927

**Published:** 2025-01-24

Mai J, Li Z, Xia X, Zhang J, Li J, Liu H, Shen J, Ramirez M, Li F, Li Z, Yokoi K, Liu X, Mittendorf EA, Ferrari M, Shen H. Adv Sci (Weinh). 2021, 8:2100166.

https://doi.org/10.1002/advs.202100166

In paragraph 1 of the “Discussion and Conclusion” section, a new reference “B. Temizoz, E. Kuroda, K. Ohata, N. Jounai, K. Ozasa, K. Kobiyama, T. Aoshi, and K.J. Ishii, Eur J Immunol. 2015, 45, 1159” is added after the text “… potentially as a result of crosstalk between the TLR and STING‐mediated pathways and m‐particle‐activated TRIF/MAVS signaling”.

In paragraph 3 of the “Discussion and Conclusion” section, a new reference “C. Aroh, Z. Wang, N. Dobbs, M. Luo, Z. Chen, J. Gao, N. Yan, J Immunol. 2017, 199, 3840” is added after the text “In line with the study, it was previous found that a nanoparticle‐based vaccine carrying a STING‐activating polymer provided better therapeutic outcome than a polyIC‐based vaccine.”

We apologize for missing these two references in the original article.

