# Supplementary material for: Synergistic Activation of Antitumor Immunity by a Particulate Therapeutic Vaccine
Source: Adv Sci (Weinh). 2021 Apr 15;8(12):2100166. doi: 10.1002/advs.202100166 (PMC8224417; doi:10.1002/advs.202100166)
Supplement: Supplementary file 1 — Supporting Information [file ADVS-8-2100166-s001.pdf]

© 2021 Wiley-VCH GmbH

ADVANCED SCIENCE
Open Access

Supporting Information

for *Adv. Sci.,* DOI: 10.1002/advs.202100166

Synergistic activation of anti-tumor immunity by a particulate therapeutic vaccine

*Junhua Mai, Zhaoqi Li, Xiaojun Xia, Jingxin Zhang, Jun Li, Haoran Liu, Jianliang Shen, Maricela Ramirez, Feng Li, Zheng Li, Xuewu Liu, Elizabeth A. Mittendorf, Mauro Ferrari, and Haifa Shen**

Supporting Information

Synergistic activation of anti-tumor immunity by a particulate therapeutic vaccine

*Junhua Mai, Zhaoqi Li, Xiaojun Xia, Jingxin Zhang, Jun Li, Haoran Liu, Jianliang Shen, Maricela Ramirez, Feng Li, Zheng Li, Xuewu Liu, Elizabeth A. Mittendorf, Mauro Ferrari, and Haifa Shen**

Dr. J. Mai, Dr. H. Liu, Ms. M Ramirez, Prof. X Liu
Department of Nanomedicine, Houston Methodist Academic Institute, Houston, Texas 77030, USA

Dr. Z. Li, Dr. J. Zhang, Dr. J. Li
Department of Nanomedicine, Houston Methodist Academic Institute, Houston, Texas 77030, USA
&
Xiangya Hospital of Central South University, Changsha, Hunan, China 410000.

Dr. X. Xia
Department of Nanomedicine, Houston Methodist Academic Institute, Houston, Texas 77030, USA
&
Department of Experimental Medicine, Sun Yat-sen University Cancer Center, State Key Laboratory of Oncology in South China, Guangzhou, China 510060

Dr. J. Shen
Department of Nanomedicine, Houston Methodist Academic Institute, Houston, Texas 77030, USA
&
School of Ophthalmology & Optometry, School of Biomedical Engineering, Wenzhou Medical University, Wenzhou, China 325035

Dr. F. Li, Prof. Z. Li
Center for Bioenergetics, Houston Methodist Academic Institute, Houston, TX 77030, USA

Prof. E. A. Mittendorf
Department of Surgery, Brigham and Women's Hospital, Boston, MA 02115, USA
&
Breast Oncology Program, Dana-Farber/ Brigham and Women's Cancer Center, Boston, MA 02115, USA

Prof. M. Ferrari
Department of Nanomedicine, Houston Methodist Academic Institute, Houston, Texas 77030, USA
Current address: Department of Pharmaceutics, School of Pharmacy, University of Washington, Seattle, WA 98195, USA

Prof. H. Shen
Department of Nanomedicine, Houston Methodist Academic Institute, Houston, Texas 77030, USA
&
Houston Methodist Cancer Center, Houston, Texas 77030, USA
&
Department of Cell and Developmental Biology, Weill Cornell Medical College, New York, NY 10065, USA
E-mail: hshen@houstonmethodist.org

Figure Legends

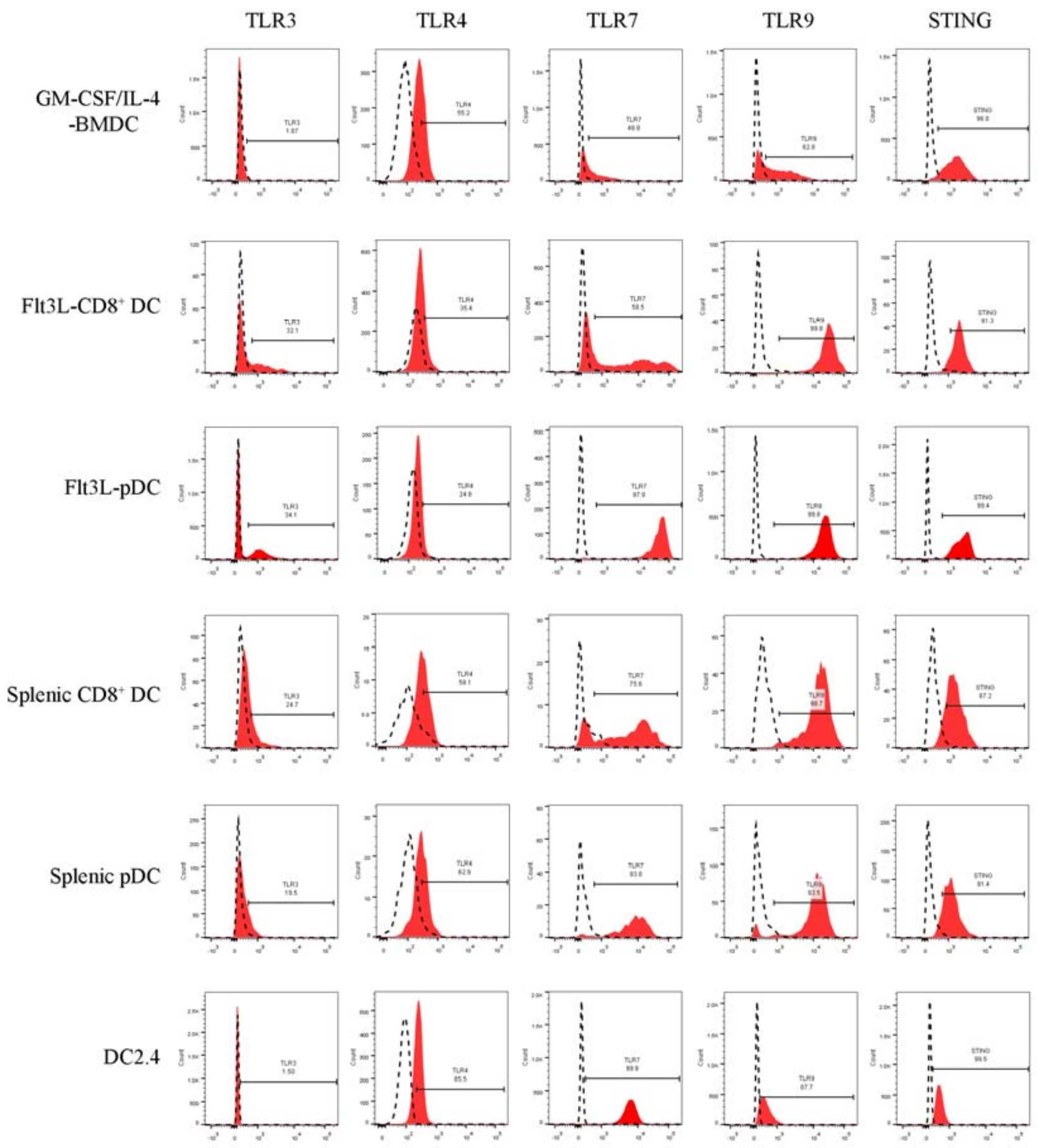

Figure S1. Flow cytometry analysis on expression of STING and TLRs in DCs. Chromatograms of unstained cells are in dashed lines, and those stained with respective antibodies are in solid red.

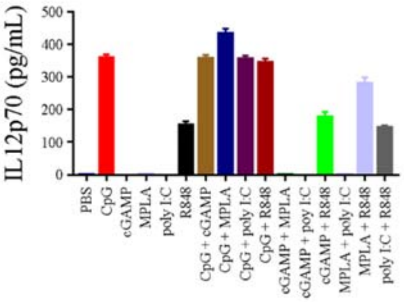

Figure S2. IL-12p70 expression by BMDCs after treatment with soluble TLR ligands and STING agonist. FLT3L-induced BMDCs were treated with a TLR ligand, a STING agonist, or their combination for 24 hours, and ELISA was applied to measure IL-12p70 levels in cell growth media. Samples were triplicated.

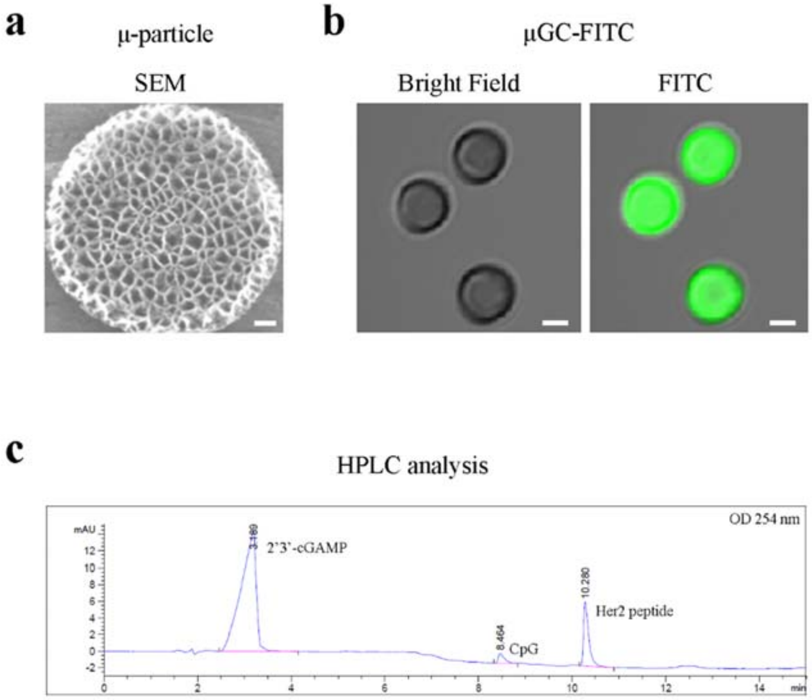

Figure S3. Empty μ-particle, liposome-loaded μ-particles and quality control. (a) SEM image of a μ-particle with 1 μm in diameter. The 40-100 nm pores in the particle can be loaded with liposomes. Scale bar: 100 nm. (b) Confocal microscopic images of μ-particles loaded with liposomes that were

packaged with FITC-CpG and cGAMP. Scale bar: 500 nm. (c) Quality control of μGCVax by HPLC. HPLC profile from μGCHer2 is shown here.

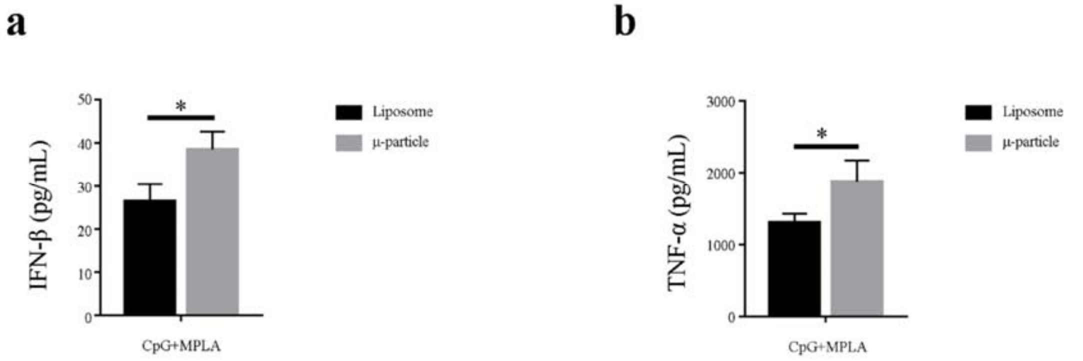

Figure S4. Comparison on stimulation of IFN-β and TNF-α expression by free liposomes and those loaded in μ-particles. BMDCs were treated with either free liposomal CpG+MPLA (labeled as "Liposome") or μ-particle-loaded CpG+MPLA (labeled as "μ-particle"), and (a) IFN-β and (b) TNF-α levels in cell growth media were measured and compared. Samples were triplicated. Statistics: Student's *t* test for comparison between two groups. *: p<0.05.

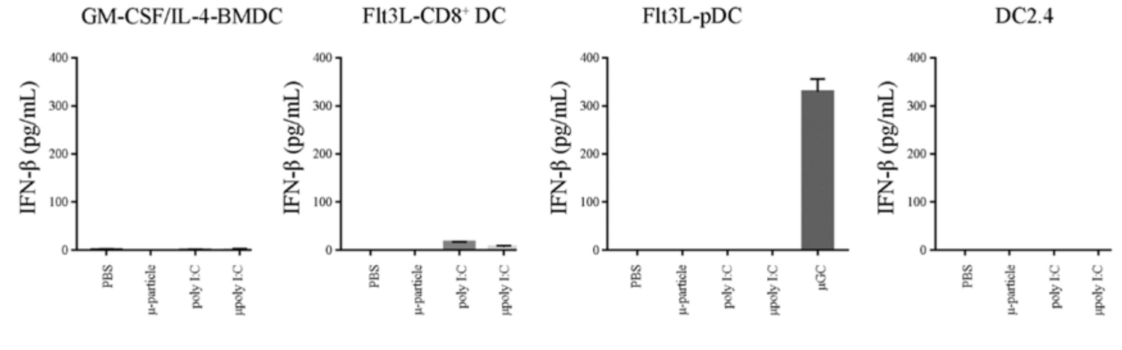

Figure S5. Lack of synergy on IFN-β expression between polyI:C and the μ-particle. GM-CSF/IL-4-induced BMDC, Flt3L-induced $CD8^+$ DC, Flt3L-induced pDC and DC2.4 cells were treated with the μ-particle, polyI:C, or polyI:C-loaded μ-particle (μpolyI:C) for 18 hours, and IFN-β

levels in cell growth media were measured. μGC served as a positive control in the Flt3L-pDC treatment study. Samples were triplicated.

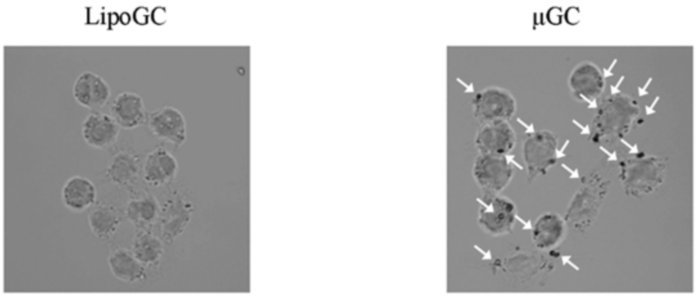

Figure S6. Microscopic views of DC2.4 cells incubated with LipoGC (left) or μGC (right). White arrows point to μGC particles.

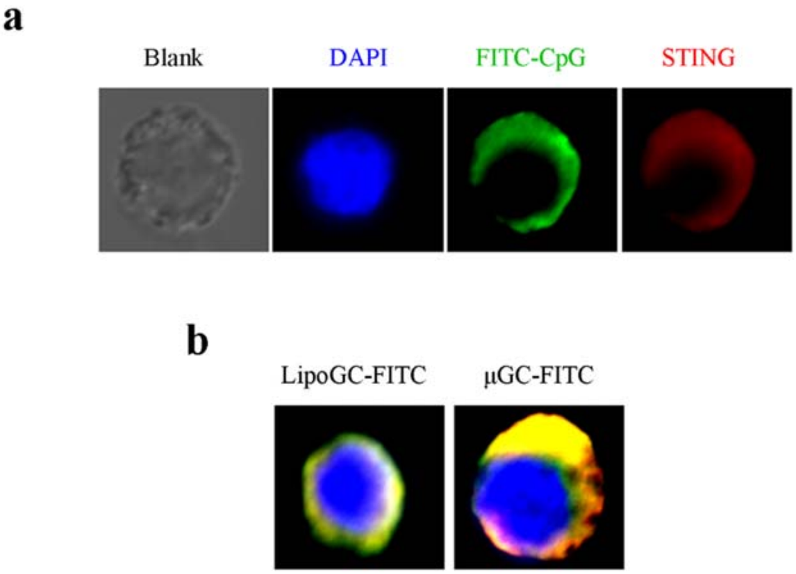

Figure S7. Enhanced STING stimulation by μGC. DC2.4 cells were treated with LipoGC-FITC or μGC-FITC, and stained with DAPI for nucleus (in blue) and anti-STING antibody (in red). (a) Representative images of DAPI, FITC-CpG, and STING staining. (b) Stronger STING activation in

cells treated with μGC-FITC than those with LipoGC is demonstrated by dense STING staining as a result of activation.

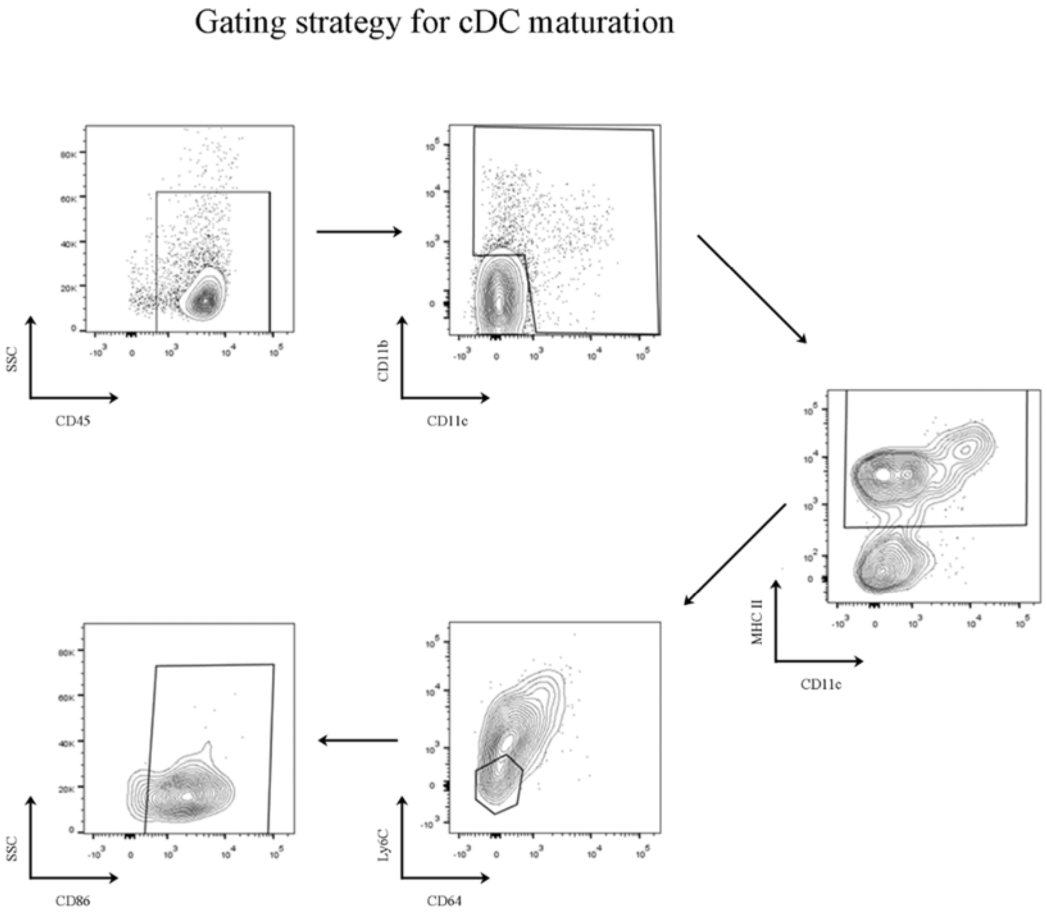

Figure S8. Schematic view on gating strategy for measurement of maturation status of cDCs.

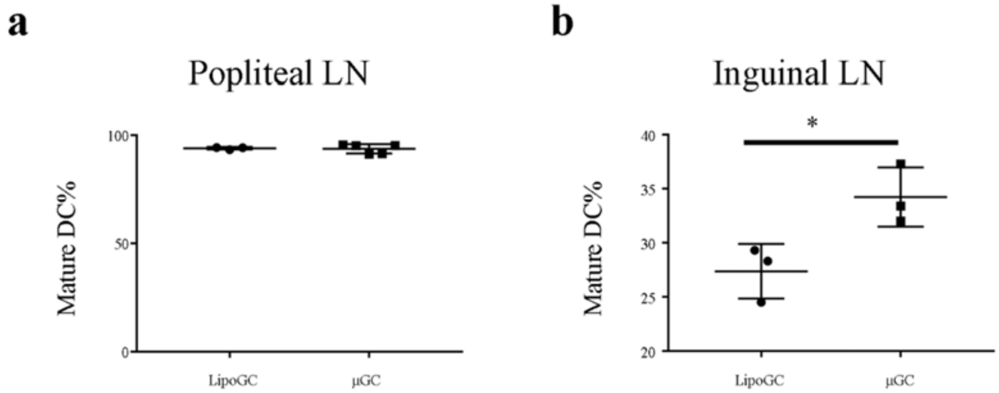

Figure S9. Flow cytometry analysis on mature DCs in lymph nodes. Samples were triplicated. Statistics: Unpaired Student's *t* test for comparison between two groups. *: $p<0.05$.

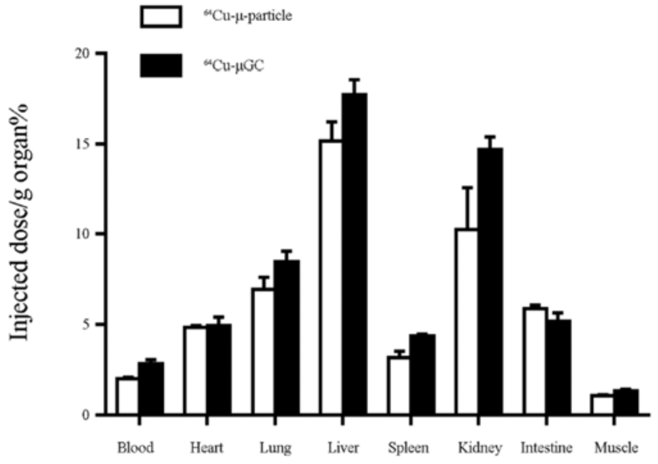

Figure S10. Biodistribution of μ-particle and μGC in major organs. BALB/c mice with primary TUBO tumors were treated with ^{64}Cu-labeled μ-particles or μGC, and radioactivity in major organs were measured 48 hours later. Samples were triplicated.

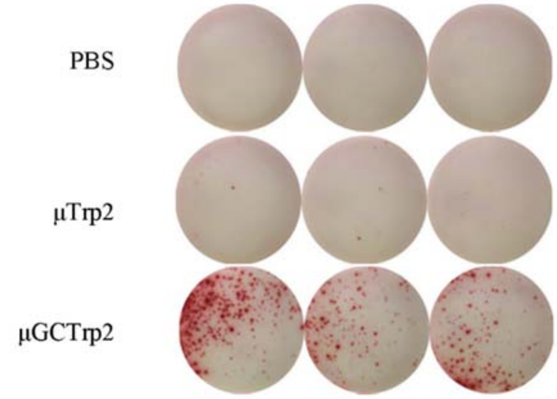

Figure S11. Images of ELISpot assay from different treatment groups.

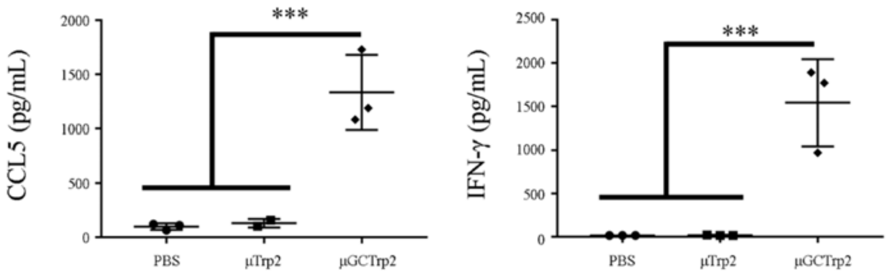

Figure S12. Cytokine production by splenic cells after stimulation with Trp2 antigen peptide in an *ex vivo* setting. Splenic cells from post-vaccination mice were seeded in 24-well plates and stimulated with 10 μg/mL Trp2 peptides in growth medium. ELISA was applied to measure cytokine levels. Samples were triplicated. Statistics: One-way ANOVA for multi-group comparison. ***: $p<0.001$.

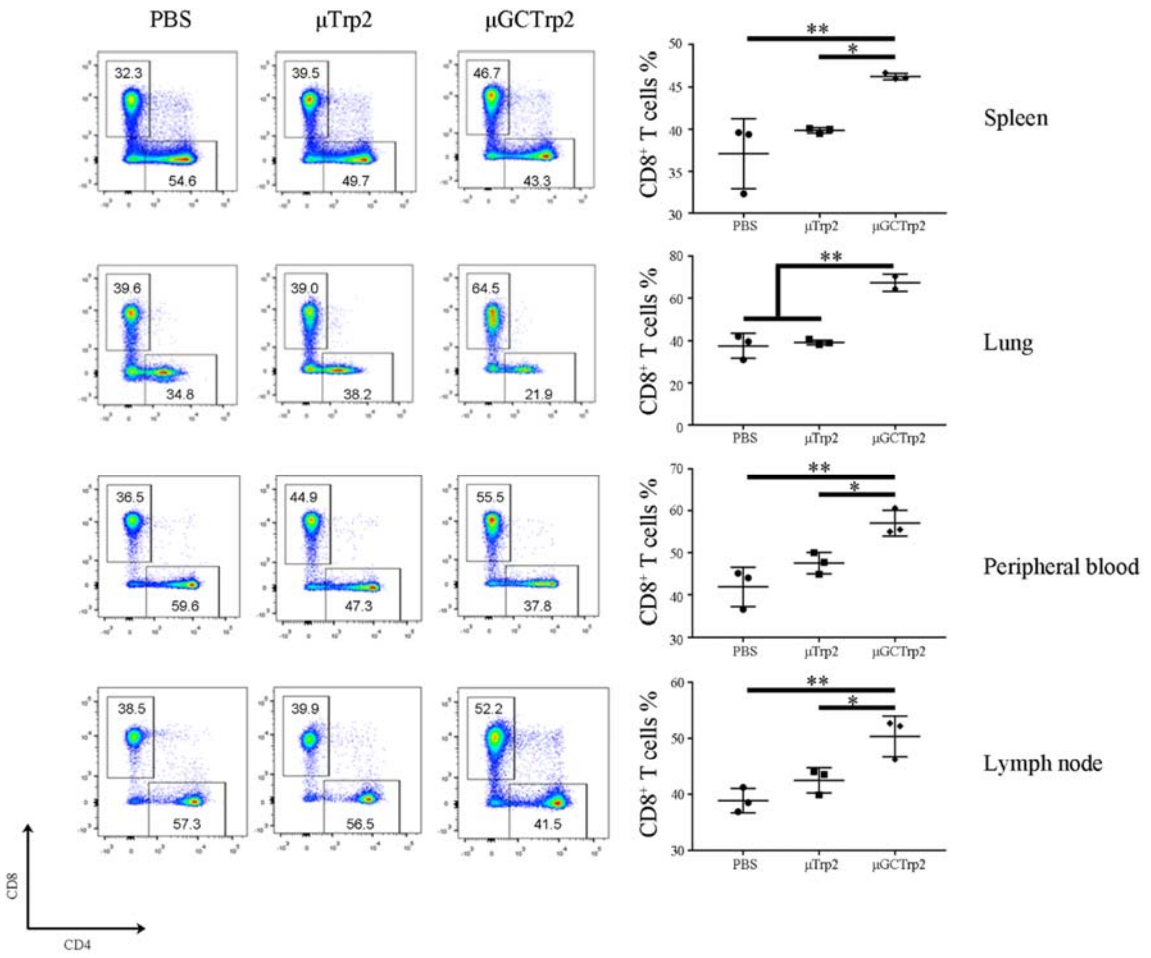

Figure S13. Representative chromatograms of flow cytometry to determine CD4$^+$ and CD8$^+$ T cells in spleens, lungs, peripheral blood and lymph nodes. Representative graphs are shown. Samples were triplicated. Statistics: One-way ANOVA for multi-group comparison. *: $p<0.05$; **: $p<0.01$.

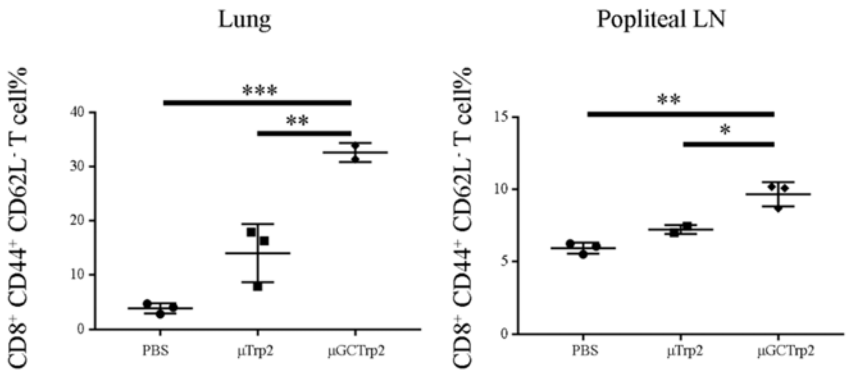

Figure S14. Effector memory T cells in tumor-bearing lungs and popliteal lymph nodes from post-vaccination mice. Effector memory T cells were determined based on CD44^{+}CD62L^{-} cell surface markers. Samples were triplicated. Statistics: One-way ANOVA for multi-group comparison. *: p<0.05; **: p<0.01.

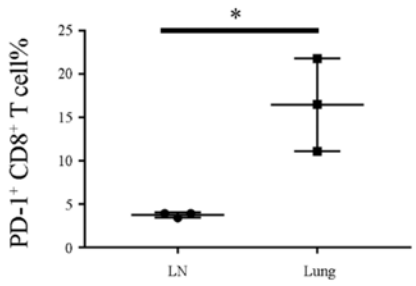

Figure S15. PD-1 expression in CD8^{+} T cells from lymph nodes and tumor-bearing lungs from µGCTrp2-treated mice. Samples were triplicated. Statistics: Student's *t* test for comparison between two groups. *: p<0.05.

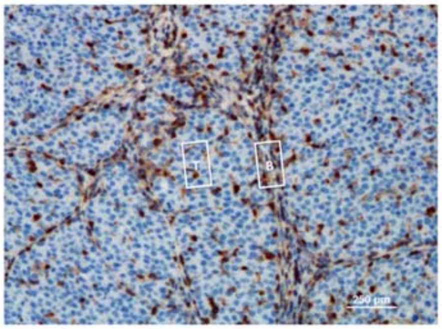

Figure S16. Regions of tumor boundary and tumor parenchyma that were used to determine T cell density. T: tumor bed; B: tumor boundary.

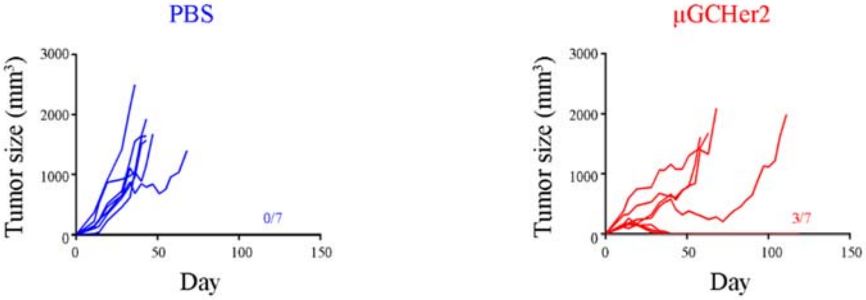

Figure S17. Growth curves of individual tumors in PBS control and μGCHer2 vaccination groups. There were 7 mice in each group. Three mice in the μGCHer2 vaccination group were tumor-free 35 days after vaccination.

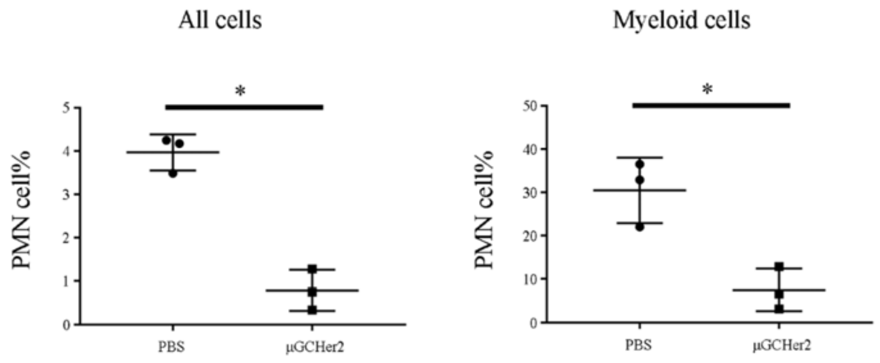

Figure S18. Changes in tumor-infiltrated myeloid cells after mice with TUBO tumors were treated with μGCHer2. Left panel: Percentage of PMN-cells in total cells inside the tumor tissue. Right panel: Percentage of PMN-cells in all $CD11b^+$ myeloid cells from the tumor tissue. Samples were triplicated. *: $p<0.05$.

Gating strategy for DC subpopulations

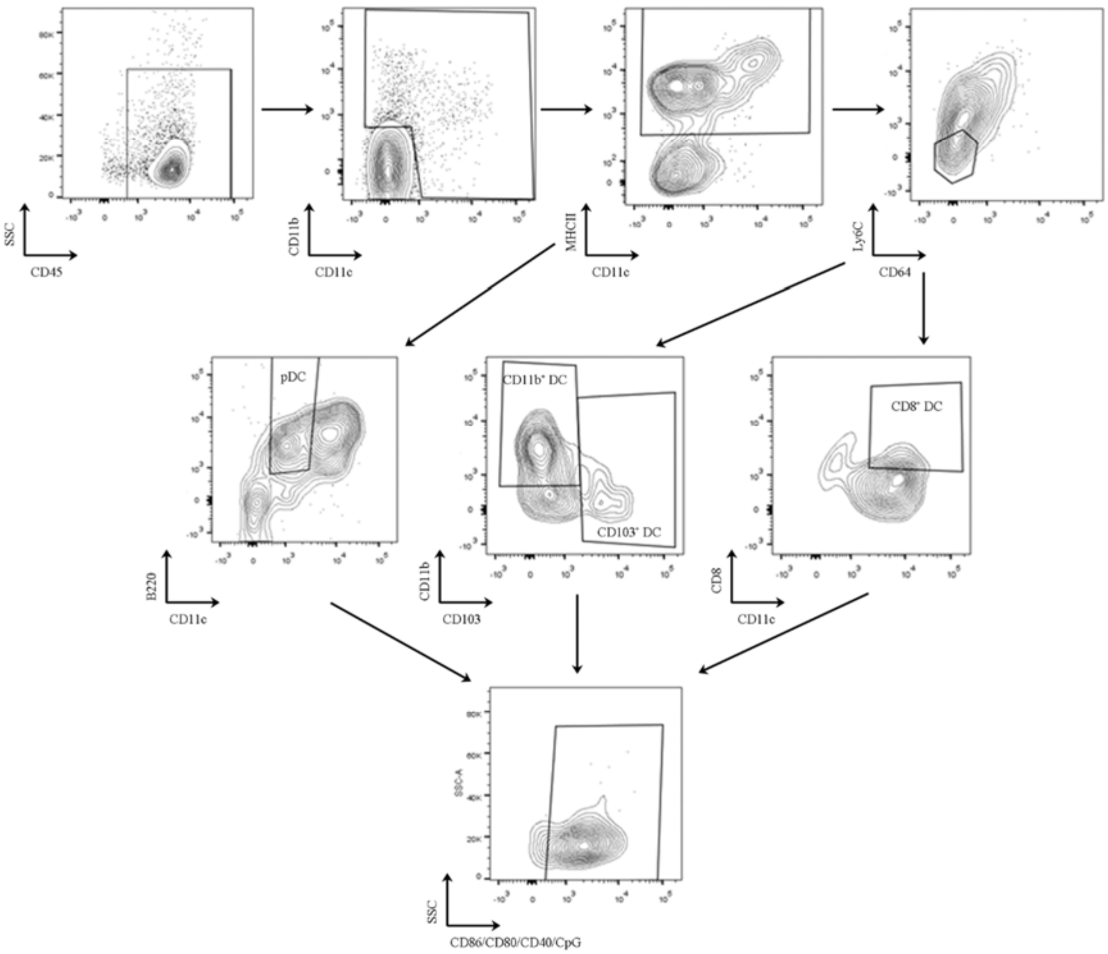

Figure S19. Schematic view on gating strategy for measurement of subpopulations of DCs with flow cytometry.

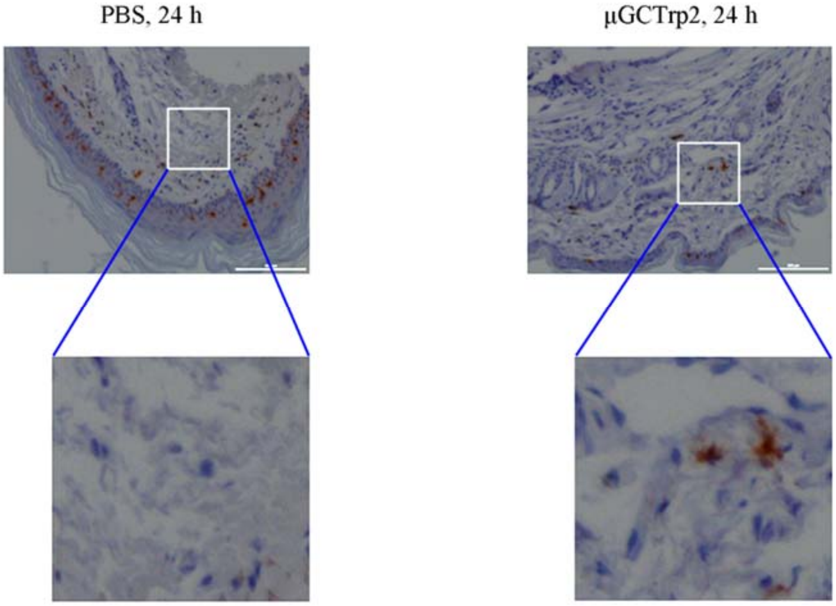

Figure S20. Particulate vaccine treatment attracted local DC migration. Foot pads in PBS control mice and μGCTrp2-treatment mice were processed, and tissue blocks were stained with an anti-CD207 antibody for Langerhans cells (in brown). Multiple Langerhans cells have exited the epidermis layer and entered the dermis region.

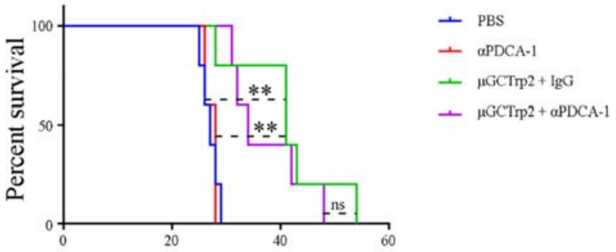

Figure S21. pDC depletion on therapeutic outcome. Mice with B16 melanoma lung metastasis were treated with μGCTrp2 with or without anti-PDCA-1 (αPDCA-1), and animal survival was compared. IgG served as a control for αPDCA-1. n = 5 mice/group. **: $p<0.01$.

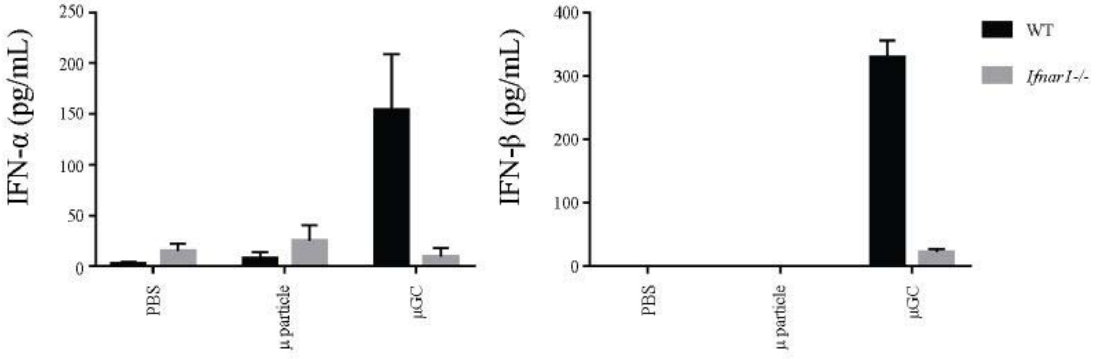

Figure S22. IFN-I expression in pDCs derived from wild-type (WT) and *Ifnar1* knockout (*Ifnar1*$^{-/-}$) mice. WT and *Ifnar1*$^{-/-}$ pDCs were treated with μ-particles or μGC, and cytokine levels in growth media were measured and compared. Samples were triplicated.
